# Comparison and multi-model inference of excess risks models for radiation-related solid cancer

**DOI:** 10.1007/s00411-022-01013-0

**Published:** 2023-01-21

**Authors:** Alberto Stabilini, Luana Hafner, Linda Walsh

**Affiliations:** 1 Swiss Federal Nuclear Safety Inspectorate ENSI, Industriestrasse 19, 5201 Brugg, Switzerland; 2 Department of Physics, University of Zurich, Winterthurerstrasse 190, 8057 Zurich, Switzerland; 3 Department of Radiation Safety and Security, Paul Scherrer Institute, Forschungsstrasse 111, 5232 Villigen PSI, Switzerland

**Keywords:** Radiation-induced cancer risk, Model comparison, Multi-model inference, Radiation epidemiology

## Abstract

In assessments of detrimental health risks from exposures to ionising radiation, many forms of risk to dose–response models are available in the literature. The usual practice is to base risk assessment on one specific model and ignore model uncertainty. The analysis illustrated here considers model uncertainty for the outcome all solid cancer incidence, when modelled as a function of colon organ dose, using the most recent publicly available data from the Life Span Study on atomic bomb survivors of Japan. Seven recent publications reporting all solid cancer risk models currently deemed plausible by the scientific community have been included in a model averaging procedure so that the main conclusions do not depend on just one type of model. The models have been estimated with different baselines and presented for males and females at various attained ages and ages at exposure, to obtain specially computed model-averaged Excess Relative Risks (ERR) and Excess Absolute Risks (EAR). Monte Carlo simulated estimation of uncertainty on excess risks was accounted for by applying realisations including correlations in the risk model parameters. Three models were found to weight the model-averaged risks most strongly depending on the baseline and information criteria used for the weighting. Fitting all excess risk models with the same baseline, one model dominates for both information criteria considered in this study. Based on the analysis presented here, it is generally recommended to take model uncertainty into account in future risk analyses.

**Supplementary Information:**

The online version contains supplementary material available at 10.1007/s00411-022-01013-0.

## Introduction

The research in radiation epidemiology has been devoting efforts for many decades, to studying the correlations of radiation exposure effects and the related risks of developing malignant diseases (RERF 2014). Such studies, including the Life Span Study (LSS) on atomic bomb survivors (e.g., Grant et al. 2017; Hsu et al. 2013; Preston et al. 2007), have thoroughly investigated several physical, biological and physiological aspects of radiation-related malignancies, with the ultimate goal of providing the most accurate model to relate ionising radiation exposures with the associated disease onset risks (Grant et al. 2017; Preston et al. 2007). The information provided by these studies is of paramount importance as it constitutes the basis of radiation protection, thus impacting activities and situations in which natural or artificial ionising radiation is involved (ICRP 2007).

Throughout the decades, the scientific community has contributed to an evolution of the models adopted to describe the risk related to ionising radiation exposures (ICRP 2007). The variety of models proposed comes, not only from the diverse approaches and techniques adopted to analyse the datasets from radiation epidemiological studies, but also, and predominantly, from the facts (a) that more studies have gradually become available with the extensive use of nuclear technologies and radiation exposure awareness, and (b) that such data-sets, being in fact observational, provide more reliable and accurate information the longer the follow-up period of the cohort.

Research groups and committees often recommend the adoption of the developed radiation exposure risk models based on their goodness of fit to the analysed epidemiological datasets, while retaining the simplest mathematical form that adequately captures trends in the data, i.e., the minimum number of parameters (Grant et al. 2017; Little et al. 2008). To this end, a number of statistical measures, such as the Akaike Information Criterion (AIC) or the Bayesian Information Criterion (BIC), have been used to give a metric, a criterion, to objectively quantify the goodness of fit, additionally taking the number of parameters into account (Akaike 1972; Schwarz 1978).

However, due to the existing non-negligible degree of uncertainty of the models proposed and the variety of epidemiological data-sets available, the choice of preferred models to assess the risk of developing cancer remains anything but a trivial task (UNSCEAR 2006). As a result, when one wishes to calculate radiation-induced cancer risks, one is typically confronted with several co-existing up-to-date models, each of which is often supported by a radiation protection body or committee, that might differ in their mathematical form and yield somewhat different assessments and associated uncertainties (BEIR 2006; ICRP 2007; UNSCEAR 2006).

From this point of view, a single preferred model for the assessment of the onset risk of a specific cancer type or category would be desirable. Statistical techniques on Multi-Model Inference (MMI) could provide such a preferred model (Zhang and Townsend 2009). The latter would facilitate risk assessment comparisons performed by different radiation protection bodies or groups and, more fundamentally, would provide a better estimate of the risk by considering the uncertainty involved in model choice, as by definition it encompasses models obtained with different approaches and data-sets (Walsh 2007). An effort in this direction was initiated by Walsh and Kaiser (2011) and continued by Kaiser et al. (2012), Walsh and Schneider (2013) and Schöllnberger et al. (2012, 2018, 2020) who applied MMI to combine either all the published models or models from a pre-defined set with the aim of obtaining a more comprehensive estimate of the radiation-related excess risk for several outcomes, also accounting for model uncertainty.

In this study, the focus is on all solid cancer risks, with the twofold objective of comparing excess risk estimates and related uncertainties of eleven currently published and recommended risk models, and of performing multi-model inference on these with the aim of building a single “composite model” obtained by averaging all the eleven starting models with due weights in order to account for model choice uncertainty. Estimation uncertainty on the excess risk provided by each model studied, including the composite model, has been assessed with Monte Carlo (MC) calculations. Since the accuracy yielded by MC is influenced by the number of realisations defined, in this piece of work a section was dedicated to presenting the results of the present investigations on this topic, which also justify the number of realisations adopted throughout the study.

## Materials and methods

### Data and software

The models were fitted to the most recent LSS solid cancer incidence data of the atomic bomb survivors, published by Grant et al. (2017) DATAFILE: https://www.rerf.or.jp/en/library/data-en/lssinc07/. As described by Grant et al. (2017), first solid cancer incidence follow-up data were collected from cities and prefecture cancer incidence registries. As the data-set focuses on solid cancers, all lymphohematopoietic malignancies, such as leukaemia, lymphoma and myeloma, were not considered. The Dosimetry System 2002 Revision 1 (DS02R1) was used for the estimation of the atomic bombing survivors exposures (Cullings et al. 2017). The weighted colon dose *D,* used to relate the risk of malignancies with radiation exposure, assumes in its calculation a value for the neutron relative biological effectiveness (RBE) of 10 relative to gammas; i.e., for each given organ, the neutron absorbed dose was multiplied by a factor 10 before being summed to the gamma absorbed dose contribution.

### Considered models

In the present study, all the published radiation-related solid cancer risk models that are known to the authors and currently deemed plausible by the scientific community were considered. In order to compare them, models were re-fitted to the most recent publicly available solid cancer incidence data of the A-bomb survivors LSS (Grant et al. 2017). Models based on mortality data were included in the analysis under the assumption that model forms developed and fitted using cancer mortality data can be directly applied and fitted to cancer incidence data. The disease models used are Poisson regression models to describe the distribution of the number of solid cancer incidence cases in a given stratum of the LSS cohort. The expected number of solid cancer cases in a stratum is given by:

1 $$PY\cdot \uplambda \left(a, e, d, s, c\right),$$

where $PY$ is the number of person-years at risk in this stratum and $\uplambda (a, e, d, s, c)$ is the cancer incidence rate in this stratum with average attained age *a*, average age at exposure *e*, average dose *d*, sex *s* and city *c*.

All solid cancer risk models have historically mainly been characterised by linear dose–response trends (BEIR 2006; ICRP 2007). However, recent analyses including the most recent follow-up data, have with due uncertainties suggested that a linear-quadratic risk dependence on the dose may need to be considered, rather than just a pure linear one (Grant et al. 2017; Ozasa et al. 2012; UNSCEAR 2006; Brenner et al. 2022). In particular, the recent works of Ozasa et al. (2012) and Brenner et al. (2022) specifically focused on investigating this non-linear dose–response hypothesis, finding upward curvatures of the excess risk with dose for both males and females when mortality data were used in the analysis, and for males only, when incidence data were considered. Though the hypothesis of a curvature in the dose–response is yet to be generally confirmed by the scientific community, its plausibility has increased as more recent LSS data have become available. For each model, it was therefore decided to implement both the linear and the linear-quadratic dose–response forms. Exceptions are the BEIRVII Phase 2 (2006) model, for which only a linear expression was published, and the model of Little et al. (2008), whose form is linear-quadratic-exponential, i.e., considering an additional term that depends on the dose exponentially. All the risk models considered have the form:

2 $$\begin{array}{c}\uplambda (a, e, d, s, c) = {\uplambda }_{0}(a, s,e,c) +\mathrm{ EAR}(a, e, d, s)\\\uplambda (a, e, d, s, c) = {\uplambda }_{0}(a, s,e,c) \cdot [1 +\mathrm{ ERR }\left(a, e, d, s\right)]\end{array}$$

where $\uplambda (a, e, d, s, c)$ is the cancer incidence rate, ${\uplambda }_{0}\left(a, s,e,c\right)$ is the baseline cancer incidence rate, and $\mathrm{EAR}(a, e, d, s)$ and $\mathrm{ERR }\left(a, e, d, s\right)$ are the radiation-related Excess Absolute Risk (EAR) and Excess Relative Risk (ERR) functions in a given stratum, respectively.

In the formulation of the excess risks, the dose–response (either linear or linear-quadratic) is multiplied by additional terms, accounting for risk modification effects, related for example to the age attained, age at exposure or sex. A summary of the models considered, including the mathematical expression of their baseline rates and excess risk and their relevant risk coefficients, is shown in Table 1.

**Table 1:** List of risk models analysed in the present study

Model	Baseline rates	Nb^1^	Excess risks	Nb^2^
BEIR VII phase 2 (2006)	RR: Stratification on sex, city, age at exposure, and attained age (500 strata) AR: $$\exp \left( \begin{gathered} k_{1,s} c + k_{2,s} \left( {\frac{e - 30}{{10}}} \right) + k_{3,s} \left( {\frac{e - 30}{{10}}} \right)^{2} + k_{4,s} \left( {\frac{e - 50}{{10}}} \right)^{2}_{e > 50} \hfill \\ + k_{5,s} \log \left( \frac{a}{70} \right) + k_{6,s} \log^{2} \left( \frac{a}{70} \right) + k_{7,s} \log^{2} \left( \frac{a}{40} \right)_{a \ge 40} \hfill \\ + k_{8,s} \log^{2} \left( \frac{a}{70} \right)_{a \ge 70} + k_{9} \left( {\frac{e - 30}{{10}}} \right)\log \left( \frac{a}{70} \right) \hfill \\ \end{gathered} \right)$$	RR: 500 AR: 9	$$\alpha_{s} D\exp \left( {\tau e^{*} } \right)\left( \frac{a}{70} \right)^{\nu } e^{*} = \left\{ {\begin{array}{*{20}c} {\begin{array}{*{20}c} {\frac{e - 30}{{10}}} & {e < 30 \,{\text{years}}} \\ \end{array} } \\ {\begin{array}{*{20}c} 0 & {e \ge 30 \,{\text{years}}} \\ \end{array} } \\ \end{array} } \right.$$	3
Grant et al. (2017)	${\text{exp}}\left( {{\text{k}}_{{\text{1,s}}} {\text{ + k}}_{{\text{2,s}}} {\text{log}}\frac{{\text{a}}}{{{70}}}{\text{ + k}}_{{\text{3,s}}} {\text{log}}^{{2}} \frac{{\text{a}}}{{{70}}}{ + }\left[ {{\text{k}}_{{\text{4,s}}} {\text{log}}^{{2}} \frac{{\text{a}}}{{{70}}}} \right]_{{\text{a > 70}}} {\text{ + k}}_{{5}} \left( {\frac{{\text{byr - 1915}}}{{{10}}}} \right){\text{ + c NIC}}} \right)$	6	$\left( {\alpha D + \beta D^{2} } \right)\exp \left( {\tau \left( {\frac{e - 30}{{10}}} \right) + \nu \ln \left( \frac{a}{70} \right) + \phi \left( {K > 4} \right)} \right)\left( {1 + \sigma s} \right)$	6
Ozasa et al. (2012)	RR: Stratification on city, sex, age at exposure and attained age (500 strata) AR:${\text{exp}}\left( {{\text{k}}_{{\text{1,s}}} {\text{ c + k}}_{{\text{2,s}}} \left( {\frac{{\text{e - 30}}}{{{10}}}} \right){\text{ + k}}_{{\text{3,s}}} \left( {\frac{{\text{e - 30}}}{{{10}}}} \right)^{{2}} {\text{ + k}}_{{\text{4,s}}} \left( {\frac{{\text{e - 30}}}{{{10}}}} \right)^{{2}}_{{\text{e > 30}}} {\text{ + k}}_{{\text{5,s}}} \left( {\frac{{\text{e - 50}}}{{{10}}}} \right)^{{2}}_{{\text{e > 50}}} {\text{ + k}}_{{\text{6,s}}} {\text{ log}}\left( {\frac{{\text{a}}}{{{70}}}} \right){\text{ + k}}_{{\text{7,s}}} {\text{ log}}^{{2}} \left( {\frac{{\text{a}}}{{{70}}}} \right){\text{ + k}}_{{\text{8,s}}} {\text{ log}}^{{2}} \left( {\frac{{\text{a}}}{{{40}}}} \right)_{{{\text{a}} \ge {40}}} {\text{ + k}}_{{\text{9,s}}} {\text{ log}}^{{2}} \left( {\frac{{\text{a}}}{{{70}}}} \right)_{{{\text{a}} \ge {70}}} {\text{ + k}}_{{{10}}} { }\left( {\frac{{\text{e - 30}}}{{{10}}}} \right){\text{log}}\left( {\frac{{\text{a}}}{{{70}}}} \right)} \right)$	RR: 500 AR: 10	$\left( {\alpha D + \beta D^{2} } \right)\exp \left( {\tau \left( {\frac{e - 30}{{10}}} \right) + \nu \ln \left( \frac{a}{70} \right) + \phi \left( {K > 4} \right)} \right)\left( {1 + \sigma s} \right)$	6
Preston et al. (2007)	${\text{exp}}\left( {k_{{\text{1,s}}} { + }k_{{\text{2,s}}} {\text{naga + }}k_{{\text{3,s}}} {\text{log}}\frac{{\text{a}}}{{{70}}}{ + }k_{{\text{4,s}}} {\text{log}}^{{2}} \frac{{\text{a}}}{{{70}}}{ + }\left[ {k_{{\text{5,s}}} {\text{log}}^{{2}} \frac{{\text{a}}}{{{70}}}} \right]_{{\text{a > 70}}} {\text{ + k}}_{{\text{6,s}}} \left( {\frac{{\text{e - 30}}}{{{10}}}} \right){ + }k_{{\text{7,s}}} \left( {\frac{{\text{e - 30}}}{{{10}}}} \right)^{{2}} {\text{ + c NIC}}} \right)$	8	$\left( {\alpha D + \beta D^{2} } \right)\exp \left( {\tau \left( {\frac{e - 30}{{10}}} \right) + \nu \ln \left( \frac{a}{70} \right) + \phi \left( {K > 4} \right)} \right)\left( {1 + \sigma s} \right)$	6
INWORKS-Leuraud (2021)	RR: Stratification on city, sex, year of birth, attained age (547 strata) AR: As in Ozasa et al. (2012)	RR: 547 AR: 10	$$\left( {\alpha D + \beta D^{2} } \right)\exp \left( {\tau \left( {\frac{e - 30}{{10}}} \right) + \nu \left( {\frac{a - 70}{{10}}} \right)} \right)\left( {1 + \sigma s} \right)$$$$\nu = \left\{ {\begin{array}{*{20}l} {\nu_{1} a < 60 \,{\text{years}}} \hfill \\ {\nu_{2} 60 < a < 80 \,{\text{years}}} \hfill \\ {\nu_{3} a > 80 \,{\text{years}}} \hfill \\ \end{array} } \right.$$	7
UNSCEAR (2006)	$\mathrm{exp}\left({\mathrm{k}}_{0}+{\mathrm{k}}_{1}\mathrm{s}+{\mathrm{k}}_{2}\mathrm{ln}\left(\frac{\mathrm{a}}{70}\right)+{\mathrm{k}}_{3}\mathrm{ln}{\left(\frac{\mathrm{a}}{70}\right)}^{2}+{\mathrm{k}}_{4}\mathrm{ln}{\left(\frac{\mathrm{a}}{70}\right)}^{3}+{\mathrm{k}}_{5}\mathrm{ln}{\left(\frac{\mathrm{a}}{70}\right)}^{4}+{\mathrm{k}}_{6}\mathrm{ln}\left(\frac{\mathrm{a}-\mathrm{e}}{40}\right) +{\mathrm{k}}_{7}\mathrm{ln}{\left(\frac{\mathrm{a}-\mathrm{e}}{40}\right)}^{2}+{\mathrm{k}}_{8}\left(\frac{\mathrm{e}-30}{10}\right)+{\mathrm{k}}_{9}{\left(\frac{\mathrm{e}-30}{10}\right)}^{2}+{\mathrm{k}}_{10}\mathrm{sln}\left(\frac{\mathrm{a}}{70}\right)+{\mathrm{k}}_{11}\mathrm{sln}{\left(\frac{\mathrm{a}}{70}\right)}^{2}+{\mathrm{k}}_{12}\mathrm{sln}{\left(\frac{\mathrm{a}}{70}\right)}^{3}+{\mathrm{k}}_{13}\mathrm{sln}\left(\frac{\mathrm{a}-\mathrm{e}}{40}\right)+{\mathrm{k}}_{14}\mathrm{sln}{\left(\frac{\mathrm{a}-\mathrm{e}}{40}\right)}^{2}+{\mathrm{k}}_{15}\mathrm{ln}\left(\frac{\mathrm{a}-\mathrm{e}}{40}\right)\mathrm{ln}\left(\frac{\mathrm{a}}{70}\right)+{\mathrm{k}}_{16}\mathrm{ln}\left(\frac{\mathrm{a}-\mathrm{e}}{40}\right)\mathrm{ln}{\left(\frac{\mathrm{a}}{70}\right)}^{2}+{\mathrm{k}}_{17}\mathrm{ln}\left(\frac{\mathrm{a}-\mathrm{e}}{40}\right)\mathrm{ln}{\left(\frac{\mathrm{a}}{70}\right)}^{3}+{\mathrm{k}}_{18}\mathrm{ln}{\left(\frac{\mathrm{a}-\mathrm{e}}{40}\right)}^{2}\mathrm{ln}\left(\frac{\mathrm{a}}{70}\right)+{\mathrm{k}}_{19}\mathrm{ln}{\left(\frac{\mathrm{a}-\mathrm{e}}{40}\right)}^{2}\mathrm{ln}{\left(\frac{\mathrm{a}}{70}\right)}^{2}+{\mathrm{k}}_{20}\left(\frac{\mathrm{e}-30}{10}\right)\mathrm{ln}\left(\frac{\mathrm{a}}{70}\right)+{\mathrm{k}}_{21}\left(\frac{\mathrm{e}-30}{10}\right)\mathrm{ln}{\left(\frac{\mathrm{a}}{70}\right)}^{2}+{\mathrm{k}}_{22}\left(\frac{\mathrm{e}-30}{10}\right)\mathrm{ ln}{\left(\frac{\mathrm{a}}{70}\right)}^{3}\right)$	22	$\left( {\alpha D + \beta D^{2} } \right)\exp \left( {\sigma s + \varepsilon \ln \left( {\frac{a - e}{{40}}} \right) + \nu \ln \left( \frac{a}{70} \right)} \right)$	5
Little et al. (2008)	As in UNSCEAR	22	$\left( {\alpha D + \beta D^{2} } \right)\exp (\delta D)\exp \left( {\sigma s + \varepsilon \ln \left( {\frac{a - e}{{40}}} \right) + \nu \ln \left( \frac{a}{70} \right)} \right)$	6

*D* represents weighted absorbed colon dose in Gy, *e* and *a* respectively indicate the age at exposure and the attained age, and *s* the sex. Risk coefficients in the baseline rate expressions are defined with the letter *k*. Risk coefficients in the excess risk forms are indicated with Greek letters. For Grant et al. (2017), Ozasa et al. (2012), INWORKS-Leuraud et al. (2021) and UNSCEAR (2006) models, the dose–response in the excess risk form reported is linear-quadratic. However, each of these models was also considered in their linear form (i.e. *β* = 0). In the linear form, the number of risk coefficients is reduced by 1. RR denotes relative risk and AR absolute risk. ${\text{c}}$ represents the city-specific effects and ${\text{NIC}}$ the not-in-city group, ${\text{byr}}$ denotes birth year, ${\text{naga}}$ represents residents in Nagasaki and ${\text{K}}$ the total shielded kerma. Indexed expressions like e.g. $\text{e>50}$ denote a threshold above which the ages are considered in the relevant expression. Nb^1^ denotes the number of risk coefficients in the baseline and Nb^2^ the number of risk coefficients in the excess risk parametrization

In the model published in the BEIR VII Phase 2 report (2006), the relative risk baseline rate function for the ERR model was handled by stratification on sex, city of exposure (Hiroshima or Nagasaki), age at exposure, and attained age, for a total of 500 parameters to estimate, as described by Pierce et al. (1996). The baseline rate function for the EAR model was modelled using the parametric model described by Preston et al. (2007). Both forms of the excess risks depend linearly on the dose, exponentially on the age at exposure, and are proportional to a certain power of the age attained. The linear dose risk coefficient α is sex-specific.

The models published by UNSCEAR (2006), with linear and linear-quadratic dose dependences, were considered. These models are based on cancer mortality data. The absolute and relative baseline rates are described by a 22-terms parametric function depending on sex (s), attained age (*a*), age at exposure (*e*) and time since exposure (*a–e*). The excess risks are also a function of the same parameters.

The LSS linear and linear-quadratic models published by Preston et al. (2007) considered in the present study are based on cancer incidence data. They feature a sex-specific parametric expression of the baseline rates, for both relative and absolute risks. The baseline rate model included city-specific (*c*) effects for the not-in-city (*NIC*) group. Both excess risks depend on sex, age at exposure and attained age. An additional term to restrict the considered dose interval to between 0 and 4 Gy was added. A dose of 4 Gy was assigned to survivors whose exposures were estimated to be larger than 4 Gy.

The analysed linear and linear-quadratic dose–response risk models proposed by Grant et al. (2017), obtained by cancer incidence data, are characterised by baseline rates for unexposed non-smokers modelled as sex-specific quadratic splines in logarithmic attained age with sex-specific log-linear trends in year of birth (byr) (i.e., age at exposure). The baseline rate model included city-specific (*c*) effects for the NIC group. The form of the excess risks is identical to the one proposed by Preston et al. (2007). The linear and linear-quadratic mortality-based dose–response risk models proposed by Ozasa et al. (2012) are similar in their mathematical formulation to the models suggested by Preston et al. (2007) and Grant et al. (2017). Their main distinction with the aforementioned models is seen in how their baseline rates were defined: in the case of the analysis of Ozasa et al. (2012), the baselines were modelled by stratification on city, sex, age at exposure and attained age (500 parameters) in their relative risk form, and by a 10-term parametric expression, based on the same set of variables, in the absolute risk form.

The models proposed by Leuraud et al. (2021), based on both the INWORKS and the LSS data-sets, feature a baseline rate modelled by stratification on city, sex, year of birth (5 years intervals), attained age (5 years interval) (547 parameters) for the relative risk and by a parametric expression, equivalent to the one described by Ozasa et al. (2012) for the absolute risk. The expression of the linear and linear-quadratic dose–response models depends also on sex, age at exposure and attained age. In particular the model adopts three different values of the parameter pertaining to the attained age risk modifier, depending on three attained age categories (< 60 years, 60–80 years, > 80 years).

The paper by Little et al. (2008) suggests the adoption of two dose–response risk models obtained from mortality data. The first, with a linear-quadratic dose dependence, is identical to the one proposed by UNSCEAR (2006) and, therefore, already accounted as such in the following analysis. The second is a variation of the linear-quadratic dose dependence of the risk, which includes in the excess risk expression an exponential third term as function of the dose. However, the risk as function of dose, obtained fitting this second model to the LSS incidence data, assumed an implausible trend, particularly at high doses. In fact, since the quadratic dose risk coefficient (β, Table 1) was negative rather than positive, and the exponential cell sterilisation coefficient (δ, Table 1) positive rather than negative, the excess risks decreased drastically with increasing dose. It was hence decided not to include this model in further analyses discussed in this study. To fit models to the data, the Epicure/AMFIT software, Version 2.00.02 (Preston et al. 1993), was used.

### Multi-model inference (MMI)

Risk model selection based on the Akaike’s information criterion (AIC) (Akaike 1972, 1978) has been already used in the radiation epidemiology field, with the purpose of giving a metric to compare different risk models and hence select the preferred one (Posada and Buckley 2004). The AIC produces a scalar, based on maximum likelihood estimates (MLEs), that summarises, based on maximum likelihood method, how well a model fits the data, relative to each of the other considered models, while considering how many parameters are used in a model (Akaike 1972). Preferable models show smaller AIC values, obtained when good agreement between the data and the prediction of the model with the fewest number of parameters occurs (Akaike 1974). Another metric that is used for model selection is the Bayesian Information Criterion (BIC) which uses a rough asymptotic approximation to the Bayes factors developed by Schwarz (Schwarz 1978). The BIC includes the number of parameters and the deviance, and it also considers the number of data points for individual data or the number of data groups or cells for binned data (Walsh 2007). Similar to the AIC, the preferable models show smaller BIC values.

Besides constituting a consolidated methodology to choose a favourite among a set of models, the AIC and BIC also create the basis for multi-model inference (MMI), where, instead of just adopting the first-ranked model for use in risk assessment, a composite model is built as a weighted combination of the existing models, whose relative weights are determined by the AIC or BIC (Posada and Buckley 2004). Consequently, the largest weight will be assigned to the preferred model, which ranked first, and therefore, will predominantly influence the composite model. The second-ranked model will have the second largest weight, and hence will have the second largest influence on the composite model, and so forth for all the models considered.

Similarly to what was proposed by Walsh and Kaiser (2011) regarding the radiation-induced leukaemia risk, in this study, the AIC was used here to combine several of the all solid cancer risk models suggested by the research community into a composite single model. However, two different approaches were considered in this study to calculate the model-averaged (composite) relative risk estimate. In a first approach the models as shown in Table 1 with the according baseline rates and excess risk parametrisation were fitted to the most recent publicly available solid cancer incidence data of the A-bomb survivors LSS (Grant et al. 2017). The resulting excess risks will be called excess risks calculated with variable baseline in the further text. In a second approach, only the baselines were fitted to the dataset to find the best fitting baseline, relative to each of the other considered baselines, with AIC. Then the best fitting baseline has been used to fit all excess risk models shown in Table 1 to the dataset in order to estimate the model-averaged excess risk only based on the comparison of the radiation risk models. These excess risks will be called excess risks with constant baseline in the further context.

The resulting composite excess risk model is obtained by a weighted average of all the *m* models illustrated above and in Table 1. For each model *i* $\in$ {1,…,*m*}, the associated normalized weight *w*_*i*_ has been calculated using Eq. (3) (Zhang and Townsend 2009) from the respective AIC values obtained from the fittings. Sets of Akaike weights were separately assigned to the ERR and EAR models.

3 $${w}_{i}=\frac{\mathrm{exp}\left(-0.5\left({\mathrm{AIC}}_{i}-\mathrm{min}\left(AIC\right)\right)\right)}{{\sum }_{j=1}^{m}\mathrm{exp}\left(-0.5\left({\mathrm{AIC}}_{j}-\mathrm{min}\left(AIC\right)\right)\right)}$$

The model-averaged (composite) ERR estimate ${\mathrm{ERR}}_{\mathrm{MMI}}$ was obtained by a weighted sum of the ERRs estimated in each of the *m* ERR models described above and in Table 1, according to Eq. (4). The same procedure was adopted to estimate the composite EAR model.

4 $${\mathrm{ERR}}_{\mathrm{MMI}}=\sum_{i=1}^{m}{w}_{i}{\mathrm{ERR}}_{i}$$

Additionally, to the AIC, the BIC and the according weights have been assessed for all models considered in the MMI in order to draw a comparison of these two metrics. The results and differences are discussed in the according sections later in the article. 

### Uncertainties

Uncertainties on the risk coefficients, expressed as 95% confidence intervals (CI), were estimated by Monte Carlo (MC) methods. Specifically, for each model, attained age, age at exposure, sex and dose value, realisations of the excess risk estimator were generated by randomly sampling the values for all the risk coefficients estimators (defining this excess risk) from their relative asymptotic joint normal distributions (i.e., accounting for their estimated covariance matrices).

It is known that the precision of any MC estimator reduces with decreasing number of realisations. Thus, for a low number of realisations, the MC estimator of any unknown quantity of interest may appreciably vary: that is, if one is to repeat the same simulation, it can substantially differ from the previously calculated one. As the number of realisations increases, the MC estimator computed from repeated simulations tends to converge, yielding equivalent results plus/minus an error, which becomes smaller the higher the number of realisations. When assessing with MC the estimation uncertainty of any excess risk coefficient the precision of the associated MC estimator will then likewise be influenced by the number of realisations. Hence, as a basis for the subsequent analyses, first the magnitudes of the model uncertainties were studied to identify a threshold in the number of realisations beyond which the MC estimator of the width of the 95% CI on the excess risk converges and does not further appreciably vary. To study how the widths of the MC estimated 95% CIs on the excess risks vary with the number of realisations, a set of simulations was carried out with increasing values of the number of realisations: 5, 10, 20, 50, 100, 200, 500, 1,000, 2,000, 5,000 and 10,000. For each number of realisations considered, MC-runs were repeated six times to obtain the average value and standard deviation of the MC estimator of the widths of the 95% CI on the excess risk. The underestimation of the standard deviation of the MC estimator arising from the limited number of samples (six runs) was corrected multiplying by a factor 2.45, obtained from the corresponding t-distribution (ISO IEC Guide 98-3 2009).

For each model, the uncertainty on the MLEs associated to the ERRs and EARs as function of dose and attained age has been expressed computing the 2.5% and the 97.5% percentiles on the 10^3^ MC-realisations. The uncertainty on the model-averaged (or composite) excess risk estimate (EAR or ERR) was estimated calculating, the 2.5% and 97.5% weighted percentiles associated to the mixture of empirical distributions of excess risk estimates obtained from all the MC risk realisations of each starting model, appropriately weighted using the corresponding Akaike and BIC weights (*w*_*i*_). This was performed for each dose and attained age in order to get the 95% CI for every excess risk estimate as function of these variables.

## Results

### Uncertainty assessment

The MC approach to assess the estimated uncertainty on an ERR, EAR or model-averaged excess risk was first investigated. Specifically, the question to answer was: what is the minimum number of MC-realisations required to be confident that the MC estimator of the width of the 95% CI on a given excess risk does not appreciably vary if one is to repeat that simulation?

**Fig. 1:**
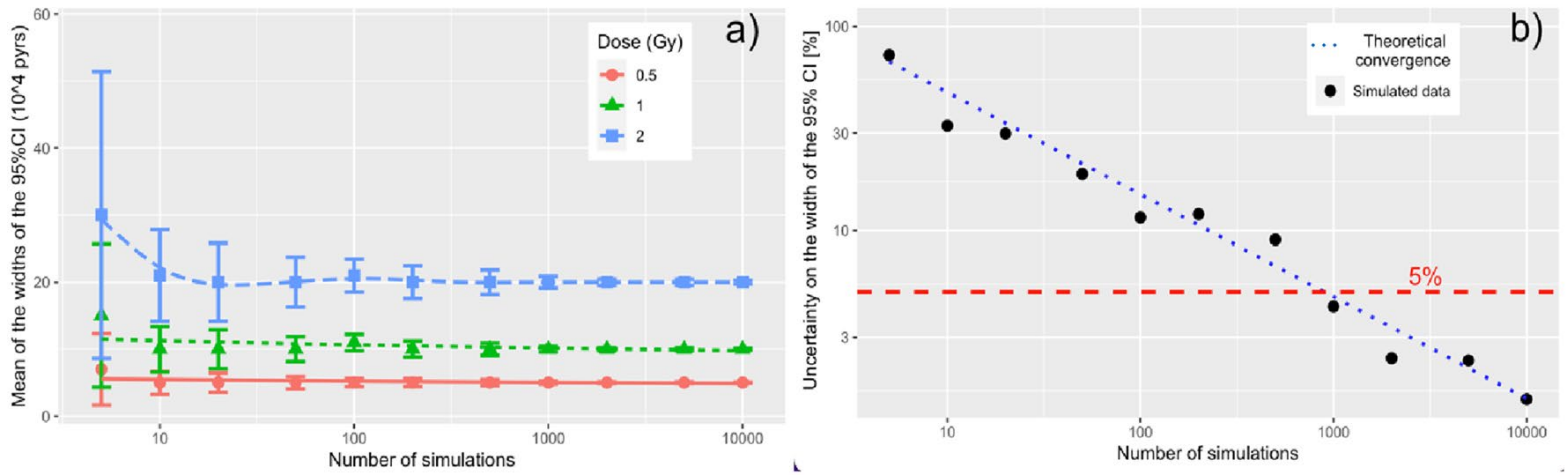
**a** Mean of the widths of the 95% CI values on the EAR realisations for women versus number of simulations, calculated using the Grant et al. (2017) linear model for different weighted colon doses of 0.5 Gy, 1 Gy and 2 Gy (attained age = 70 years, age at exposure 30 years). The error bars represent the standard error of the widths of the 95% CI excess risk. **b** Uncertainty of the estimated 95% CI excess risk values versus number of simulations. Black points indicate the relative standard error of the 1 Gy dataset illustrated in **a**. The dotted blue and the dashed red lines, respectively, show the theoretical MC- convergence, proportional to *N*^*−½*^, and the number of simulation threshold above which an uncertainty on the CI error is kept below 5% (colour figure online)

As an example, Fig. 1a shows the six-runs average estimated value and estimated uncertainty of the 95% CI width of the excess risk, using the linear Grant et al. (2017) excess absolute risk model (EAR), considering an attained age of 70 years and an age at exposure of 30 years. The uncertainties were acquired using the procedure described above, once the model was fitted with the Epicure/AMFIT software, for three different doses: 0.5 Gy (red dots), 1 Gy (green triangles) and 2 Gy (blue squares). Error bars represent the standard deviation of the uncertainty on the width of the 95% CI, obtained from the six MC-runs at each number of realisations investigated.

According to Fig. 1a, for a number of simulations < 100, one realises that MC can on average overestimate the 95% CI width of the excess risk. Besides, the variability of the MC estimator of the quantity of interest experienced for such limited number of realisations is extremely large, implying that any single MC-run can in principle yield quite a different value. As the number of simulations increases, the average estimated value of the 95% CI width stabilises for all the three doses considered, with the error bars seen to progressively reduce and becoming negligible beyond 10^3^ realisations. The latter statement is also supported by Fig. 1b, where the relative uncertainty on the dose-averaged 95% CI values (black dots) is plotted against the number of realisations considered. With the aid of this plot, one can set a threshold in the number of simulations required to keep the uncertainty on the 95% CI values below a specific target.

In this work, a relative uncertainty on the 95% CI values < 5% (represented by the dashed red line in Fig. 1b) was chosen, implying therefore the need to run 10^3^ simulations for each model, dose value and attained age considered. The threshold at 10^3^ realisations is also in agreement with the theoretical MC convergence ∝ $1/\sqrt{N}$, where *N* is the number of realisations, represented by the dotted blue line. As can be noted in Fig. 1a, b, a further increase in the numbers of simulations would yield only a limited improvement in the reduction of uncertainties, while leading to an inconvenient dilation of MC-calculation times.

### Model comparison

#### Risks as function of dose

**Fig. 2:**
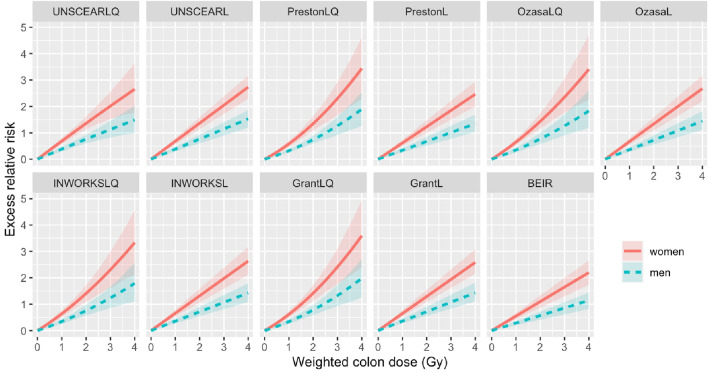
Sex-specific excess relative risks (ERRs) for the models fitted with the variable baseline as function of weighted colon dose for each model considered in this study, calculated at an attained age of 70 years and age at exposure of 30 years. Shaded areas represent 95% confidence intervals (CIs)

Figure 2 illustrates the comparison of the estimated ERR model trends which were calculated with variable baselines as function of the weighted colon dose. A list of the values found for each risk coefficient for the models shown in Table 1 after fitting is given in Table 6, in the Appendix. The comparison is presented for each sex: solid red lines indicate estimated ERRs for women, whereas dashed blue lines identify the ERR trends of males. The attained age was fixed at 70 years and the age at exposure at 30 years. The shadowed areas represent the 95% CI, obtained by the MC-calculations. With the exception of the BEIR VII Phase 2 (2006) model, all the models are presented in their linear (L) and linear-quadratic (LQ) dose-dependence formulation. Below 1.5 Gy, little deviation (< 6%) of the LQ models from their respective L-models is noticed.

The best fitting baseline to the Grant et al. (2017) dataset has been found to be the baseline from the PrestonL model. All excess risk models have therefore been fitted additionally with the PrestonL baseline parametrisation. A list of the resulting values for the risk coefficients is given in Table 7 in the Appendix. For the estimated ERR models, fitted with the PrestonL baseline, the curves are very similar to the estimated ERR-total models and shown in the Appendix (Fig. 6).

In the dose range below 1.5 Gy, all the models considered predict similar risks as function of the dose, as can be inferred comparing the resulting ERRs in Table 2, which shows average estimated ERRs (with standard deviations) at 1 Gy for the models with variable baselines of 0.64 ± 0.04 and 0.35 ± 0.02 and for the models with constant baseline of 0.59 ± 0.05 and 0.32 ± 0.03 for women and men, respectively. At higher doses (above 1.5 Gy), where the discrepancies among the models and the dose dependences become more evident, one can appreciate the less pronounced influence of the quadratic term in the UNSCEAR (2006) model compared to the others, only slightly deviating from linearity even at higher doses ($\beta /\alpha \approx {10}^{-2}$).

**Table 2:** Estimation of excess relative risk (ERR) at 1 Gy, for each model and sex, with Monte Carlo simulated confidence intervals (95% CI) in brackets

Model	Sex	ERR with variable baseline (95% CI)	ERR with constant baseline (95% CI)	Model	Sex	ERR with variable baseline (95% CI)	ERR with constant baseline (95% CI)
GrantL	Men	0.36 (0.28;0.45)	0.34 (0.26;0.43)	GrantL	Women	0.65 (0.54;0.77)	0.62 (0.50;0.74)
GrantLQ	Men	0.34 (0.26;0.42)	0.32 (0.24;0.41)	GrantLQ	Women	0.61 (0.50;0.74)	0.58 (0.47;0.69)
BEIR	Men	0.29 (0.20;0.36)	0.27 (0.19;0.34)	BEIR	Women	0.55 (0.43;0.67)	0.51 (0.40;0.61)
UNSCEARL	Men	0.38 (0.30;0.46)	0.36 (0.28;0.44)	UNSCEARL	Women	0.68 (0.57;0.79)	0.64 (0.54;0.75)
UNSCEARLQ	Men	0.38 (0.30;0.47)	0.35 (0.27;0.44)	UNSCEARLQ	Women	0.69 (0.57;0.80)	0.63 (0.53;0.75)
OzasaL	Men	0.36 (0.28;0.45)	As in GrantL	OzasaL	Women	0.67 (0.55;0.79)	As in GrantL
OzasaLQ	Men	0.35 (0.26;0.44)	As in GrantLQ	OzasaLQ	Women	0.65 (0.54;0.78)	As in GrantLQ
INWORKSL	Men	0.36 (0.27;0.45)	0.32 (0.24;0.41)	INWORKSL	Women	0.66 (0.53;0.79)	0.59 (0.47;0.72)
INWORKSLQ	Men	0.34 (0.26;0.44)	0.30 (0.22;0.39)	INWORKSLQ	Women	0.64 (0.51;0.77)	0.56 (0.43;0.69)
PrestonL	Men	0.34 (0.26;0.42)	As in GrantL	PrestonL	Women	0.62 (0.50;0.74)	As in GrantL
PrestonLQ	Men	0.32 (0.26;0.38)	As in GrantLQ	PrestonLQ	Women	0.58 (0.47;0.69)	As in GrantLQ

Age at exposure of 30 years and attained age of 70 years

**Fig. 3:**
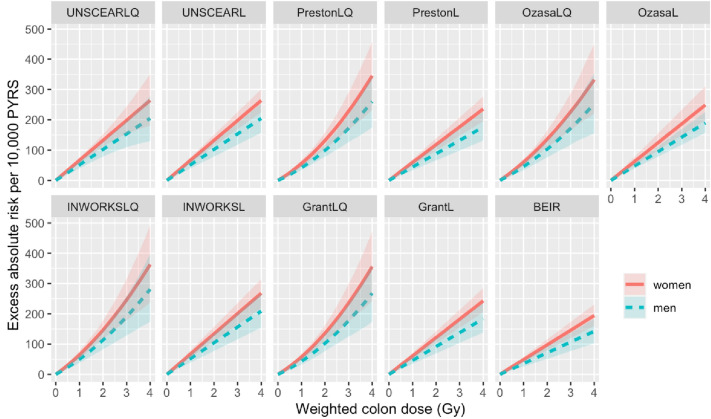
Sex-specific excess absolute risks (EARs) per 10^**4**^ person years (PYRS) as function of weighted colon dose for each model fitted with the variable baseline considered in this study, calculated at an attained age of 70 years and age at exposure of 30 years. Shaded areas represent 95% confidence intervals (CIs)

Concerning the EAR, dose–response shapes similar to the ERR findings can be inferred from the graphs in Fig. 3, where the sex-specific estimated EAR per 10^4^ PYRS for the models fitted with the variable baselines as function of the weighted colon dose is reported for each model investigated. The according figure of the estimated EAR per 10^4^ PYRS for the models fitted with the constant baseline is shown in the Appendix in Fig. 7. Dashed blue lines represent the estimated EAR-model trends for males and solid red lines the ones for women. Also in the case of EARs, for doses < 1.5 Gy, only small discrepancies among the L- and LQ-models are observed (< 7%); the EAR estimates at a dose of 1 Gy (*a* = 70, *e* = 30) reported in Table 3 are in agreement, with average estimated EARs (and standard deviations) calculated with the estimated EAR from all models of Table 3 of 61 ± 5 per 10^4^ PYRS and 46 ± 5 per 10^4^ PYRS for the models fitted with variable baseline for women and men, respectively. The according EAR estimates for the models fitted with constant baseline are 59 ± 6 per 10^4^ PYRS and 44 ± 5 per 10^4^ PYRS for the models for women and men, respectively. As noticed for the ERRs, EAR models fitted with variable baseline at doses larger than 2 Gy exhibit larger variability, with the UNSCEARLQ model being fairly close to linearity ($\beta /\alpha \approx 6\cdot {10}^{-4}$).

**Table 3:** Estimation of excess absolute risk (EAR) per 10^4^ PYRS at 1 Gy, for each model and sex, with Monte Carlo simulated confidence intervals (95% CI) in brackets

Model	Sex	EAR (95% CI) with variable baseline (95% CI)	EAR (95% CI) with constant baseline (95% CI)	Model	Sex	EAR (95% CI) with variable baseline (95% CI)	EAR (95% CI) with constant baseline (95% CI)
GrantL	Men	46 (35;59)	44 (33;56)	GrantL	Women	61 (51;71)	59 (49;69)
GrantLQ	Men	43 (32;56)	42 (31;54)	GrantLQ	Women	58 (48;68)	56 (46;67)
BEIR	Men	36 (26;46)	33 (24;43)	BEIR	Women	49 (40;58)	46 (38;55)
UNSCEARL	Men	51 (39;63)	48 (36;60)	UNSCEARL	Women	66 (56;75)	63 (53;73)
UNSCEARLQ	Men	51 (39;63)	47 (34;59)	UNSCEARLQ	Women	66 (56;76)	62 (52;72)
OzasaL	Men	47 (39;56)	As in GrantL	OzasaL	Women	62 (50;78)	As in GrantL
OzasaLQ	Men	46 (34;58)	As in GrantLQ	OzasaLQ	Women	60 (50;70)	As in GrantLQ
INWORKSL	Men	52 (39;67)	48 (36;63)	INWORKSL	Women	67 (55;79)	64 (52;76)
INWORKSLQ	Men	50 (38;65)	45 (33;58)	INWORKSLQ	Women	64 (52;77)	60 (49;58)
PrestonL	Men	44 (33;57)	As in GrantL	PrestonL	Women	59 (49;69)	As in GrantL
PrestonLQ	Men	42 (34;50)	As in GrantLQ	PrestonLQ	Women	56 (45;66)	As in GrantLQ

Age at exposure of 30 years and attained age of 70 years

#### Risks as function of attained age

**Fig. 4:**
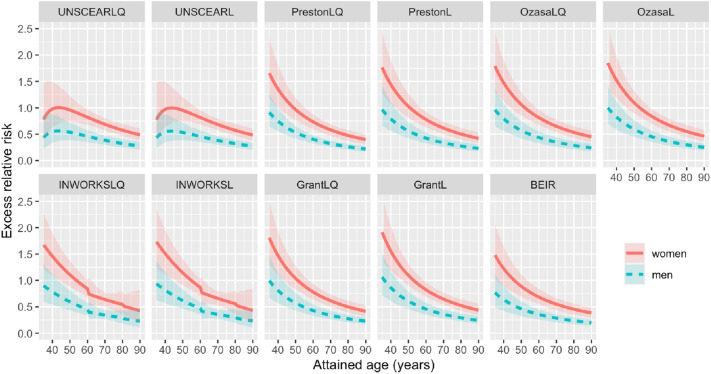
Sex-specific excess relative risks (ERRs) for the models fitted with the variable baseline as function of the attained age for each model considered in this study, assuming a weighted colon dose of 1 Gy. Shaded areas represent 95% confidence intervals (CIs)

**Fig. 5:**
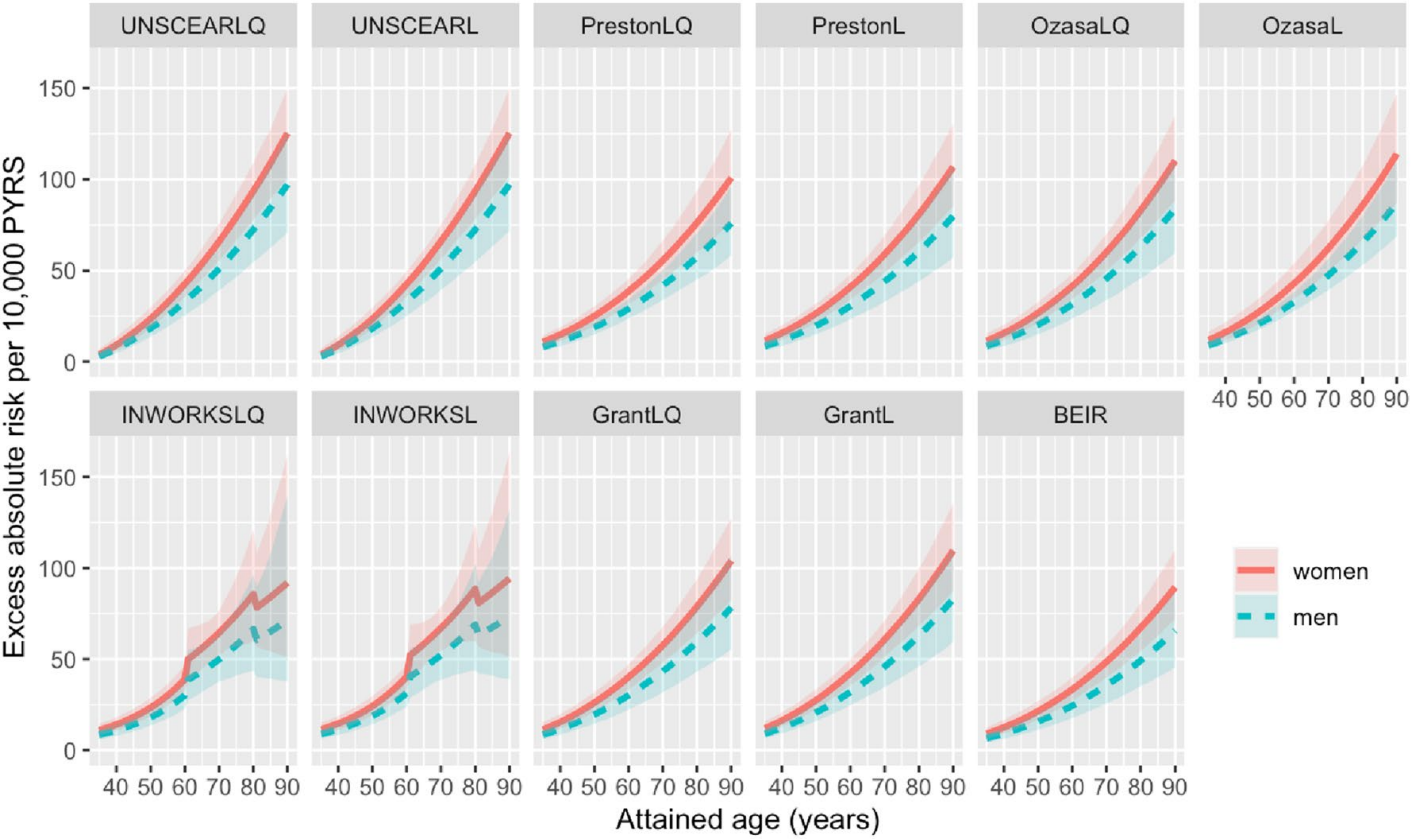
Sex-specific excess absolute risks (EARs) per 10^4^ person years (PYRS) for the models fitted with the variable baseline as function of the attained age for each model considered in this study, assuming a weighted colon dose of 1 Gy. Shaded areas represent 95% confidence intervals (CIs)

In Fig. 4 (ERR) and Fig. 5 (EAR), the comparison of the models fitted with variable baselines as function of attained age, assuming a dose of 1 Gy and an age at exposure of 30 years, is illustrated. The according figures for models fitted with the PrestonL baseline are shown in Fig. 8 (ERR) and Fig. 9 (EAR) in the Appendix. Within each panel, red solid curves indicate the ERR trend for women, whereas blue dashed lines reproduce the one for men. Shadowed areas indicate the 95% CI. Concerning ERRs (Fig. 4 and 8), albeit the general trend follows a reduction on the risk with increasing attained age, a clear difference between the UNSCEAR models and the rest of the models can be appreciated at attained ages in the range 30–50 years. This difference is a consequence of the mathematical expression of the UNSCEAR models, developed using a different data set, which has a term modelling the time since exposure *a*–*e*. According to UNSCEAR models, the ERR gradually increases in the first 10–15 years following the exposure, before experiencing a reduction similarly to other models, when the time since exposure exceeds 20 years. Another evident feature in the diagrams in Figs. 4 and 8 is the presence of discontinuities observable in the INWORKS-Leuraud models. The reason for these discontinuities straightforwardly follows from the expression of the excess risk. In fact, as described above, the risk coefficient related to attained age is assumed to take three different values according to the defined attained age intervals, to provide a better fit of the data (Leuraud et al. 2021). Discontinuities are therefore expected at the boundaries of these intervals.

Figures 5 and 9 predictably report an increasing behaviour of the EAR with attained age. Besides the aforementioned discontinuities observed in the INWORKS-Leuraud (2021) model, it should be noted that the UNSCEAR (2006) models generally present higher risks with increasing attained age, as compared to the other models that yield comparable excess risks. It is also noteworthy that UNSCEAR (2006) models indicate no excess absolute risk at the time of exposure (30 years), whereas all the other models display a positive EAR-offset. This behaviour of the UNSCEAR (2006) model can once more be attributed to the term related with the time since exposure *a*–*e*.

### Multi-model inference

#### Weights of the models with variable baseline

Table 4 summarises the associated AICs and corresponding weights, normalised separately for ERR and EAR, for all the considered models. Considering the AIC, weights assigned to the EARs span several orders of magnitude, with the UNSCEARL and -LQ ones being by far larger than the others for the models with variable baseline. According to the AIC values, the UNSCEARL and -LQ models ought to be preferred over the others comprised in this analysis. Furthermore, the model-averaged (or composite) EAR estimated as weighted sum of the EARs estimated from all the considered models, is essentially constituted solely by a combination of the UNSCEARL and -LQ models, since the weights of the other models are negligible. Similar results are obtained for the ERRs, where the range of the assigned weights extends across even larger orders of magnitude. The largest weights are assigned to the UNSCEARL and -LQ models: 0.731 and 0.269 for the EAR, and 0.728 and 0.272 for ERR, respectively.

**Table 4:** AIC values, BIC values and estimated normalized weights for each model considered in the study for excess relative risk (ERR) and excess absolute risk (EAR) separately

	EAR variable baseline				ERR variable baseline			
Model	AIC	Weight AIC	BIC	Weight BIC	AIC	Weight AIC	BIC	Weight BIC
BEIR	57,440	1.39⋅10^–13^	57,651	1.92⋅10^–9^	57,671	1.09⋅10^–15^	62,775	0
GrantL	57,439	2.45⋅10^–13^	57,611	0.96	57,438	1.51⋅10^–15^	57,610	0.98
GrantLQ	57,436	8.67⋅10^–13^	57,619	2.16⋅10^–2^	57,436	3.12⋅10^–15^	57,619	1.27⋅10^–2^
INWORKSL	57,417	1.15⋅10^–8^	57,701	2.90⋅10^–20^	57,751	1.53⋅10^–83^	63,363	0
INWORKSLQ	57,416	1.69⋅10^–8^	57,711	2.70⋅10^–22^	57,751	1.30⋅10^–83^	62,767	0
OzasaL	57,410	4.40⋅10^–7^	57,674	2.78⋅10^–14^	57,672	2.38⋅10^–66^	62,787	0
OzasaLQ	57,409	5.64⋅10^–7^	57,683	2.26⋅10^–16^	57,672	2.11⋅10^–66^	62,797	0
PrestonL	57,417	1.35⋅10^–8^	57,620	1.34⋅10^–2^	57,417	5.84⋅10^–11^	57,620	9.52⋅10^–3^
PrestonLQ	57,414	5.07⋅10^–8^	57,627	3.18⋅10^–4^	57,415	1.33⋅10^–10^	57,628	1.37⋅10^–4^
UNSCEARL	57,381	0.731	57,655	2.92⋅10^–10^	57,370	0.728	57,644	4.77⋅10^–8^
UNSCEARLQ	57,383	0.269	57,667	6.79⋅10^–13^	57,372	0.272	57,656	1.13⋅10^–10^

	EAR constant baseline				ERR constant baseline			
Model	AIC	Weight AIC	BIC	Weight BIC	AIC	Weight AIC	BIC	Weight BIC
BEIR	57,413	0.652	57,605	0.99	57,413	0.651	57,605	0.94
GrantL	57,417	0.0698	57,620	6.70⋅10^–4^	57,417	0.0803	57,620	7.34⋅10^–4^
GrantLQ	57,415	0.262	57,627	1.59⋅10^–5^	57,415	0.182	57,628	1.05⋅10^–5^
INWORKSL	57,426	0.00112	57,648	4.29⋅10^–10^	57,421	0.0101	57,644	3.68⋅10^–9^
INWORKSLQ	57,422	0.00531	57,701	1.33⋅10^–21^	57,420	0.0217	57,653	4.99⋅10^–11^
UNSCEARL	57,422	0.0065	57,614	9.87⋅10^–3^	57,418	0.0391	57,611	5.67⋅10^–2^
UNSCEARLQ	57,423	0.00306	57,626	2.94⋅10^–5^	57,420	0.0155	57,623	1.42⋅10^–4^

*AIC* Akaike information criterion; *BIC* Bayesian information criterion

Considering the BIC (also shown in Table 4), the models contributing to the MMI change. For the EAR, the models with non-negligible weights (> 0.01) are the GrantL, GrantLQ and the PrestonL models, while for ERR, only the GrantL and GrantLQ models give non-negligible contribution to the MMI. For both excess risks, the main contribution comes from the GrantL model (0.96: EAR and 0.98: ERR). ERR models with stratified baselines were all assigned with a weight of 0, confirming the disadvantage in model selection related to the large number of parameters.

#### Weights of the models with constant baseline

Using the same baseline for all models and only varying the radiation excess risk models in the fitting process results in AIC and BIC values of the same order of magnitude. Further, radiation ERR models with previously stratified baselines can now compete with the ERR models with parametrized baselines, because the number of parameters in the baseline is now constant over all models. In Table 4, the AIC, BIC and the according weights of the ERR and EAR estimated with the PrestonL baseline are shown. Considering the AIC, the contributing weights to the MMI for the EAR are those from the BEIR, GrantL and GrantLQ models with the main contribution coming from the BEIR model (weight: 0.652). Using the BIC, the BEIR model is the only model that contributes to the MMI with a weight of 0.99. Considering the AIC for the ERR, the weights are all of the same order of magnitude and no model has a weight smaller than 0.01. Therefore, all models contribute to the MMI with the main contribution coming from the BEIR model with a weight of 0.651. Considering the BIC for the ERR, only the BEIR and the UNSCEARL models contribute to the MMI with the main contribution coming from the BEIR model (weight: 0.94).

#### Estimation of model-averaged (or composite) excess risks

In Table 5, the model-averaged (composite) excess risk estimates are shown with weights based on AIC and BIC for the models with variable and constant baseline. The weights were recalculated by only considering the models with non-negligible contribution.

**Table 5:** Model-averaged (composite) excess risk estimates with weights based on AIC and BIC for the models with variable and constant baseline (BL)

Model-averaged excess risk estimates with weights based on AIC	
Variable BL	${\mathrm{EAR}}_{\mathrm{MMI}}^{\mathrm{var}}=0.73\cdot \mathrm{UNSCEARL}+0.27\cdot \mathrm{UNSCEARLQ}$
Constant BL	${\mathrm{EAR}}_{\mathrm{MMI}}^{\mathrm{const}}=0.67\cdot \mathrm{BEIR}+0.24\cdot \mathrm{GrantLQ}+0.09\cdot \mathrm{GrantL}$
Variable BL	${\mathrm{ERR}}_{\mathrm{MMI}}^{\mathrm{var}}=0.73\cdot \mathrm{UNSCEARL}+0.27\cdot \mathrm{UNSCEARLQ}$
Constant BL	${\mathrm{ERR}}_{\mathrm{MMI}}^{\mathrm{const}}=0.65\cdot \mathrm{BEIR}+0.18\cdot \mathrm{GrantLQ}+0.08\cdot \mathrm{GrantL}+0.04\cdot \mathrm{UNSCEARL}+0.02\cdot \mathrm{UNSCEARLQ}+0.02\cdot \mathrm{INWORKSLQ}+0.01\cdot \mathrm{INWORKSL}$

Model averaged excess risk estimates with weights based on BIC	
Variable BL	${\mathrm{EAR}}_{\mathrm{MMI}}^{\mathrm{var}}=0.97\cdot \mathrm{GrantL}+0.02\cdot \mathrm{GrantLQ}+0.01\cdot \mathrm{PrestonL}$
Constant BL	${\mathrm{EAR}}_{\mathrm{MMI}}^{\mathrm{const}}=\mathrm{BEIR}$
Variable BL	${\mathrm{ERR}}_{\mathrm{MMI}}^{\mathrm{var}}=0.99\cdot \mathrm{GrantL}+0.01\cdot \mathrm{GrantLQ}$
Constant BL	${\mathrm{ERR}}_{\mathrm{MMI}}^{\mathrm{const}}=0.95\cdot \mathrm{BEIR}+0.05\cdot \mathrm{UNSCEARL}$

The weights were recalculated by only considering the models with non-negligible contribution from Table 4

*AIC* Akaike information criterion; *BIC* Bayesian Information Criterion; *EAR* excess absolute risk; *ERR* excess relative risk; *MMI* multi-model inference

## Discussion

Despite lying within the same order of magnitude and showing generally similar trends, appreciable differences in the estimated excess risks can be noticed among the examined models, reported in Figs. 2, 3, 4, 5. The reason resides not only in their different mathematical form, but also due to the fact that in many cases they have originally been derived using different sets of data; for example, different follow-up time as in Preston et al. (2007) and Grant et al. (2017), mortality data rather than incidence as in UNSCEAR (2006) and Ozasa et al. (2012), or even considering different cohorts as in Leuraud et al. (2021).

As shown in Table 5, the model average excess risks have different forms when BIC weights are used instead of AIC weights. This raises the question which information criteria should be used for the purpose of model averaging. Monte Carlo simulations indicated that the AIC tends to favour models which have more parameters than the true model (Kass and Raftery 1995). This can be observed in Table 5 considering the models with the variable baselines weighted with AIC weights. For both excess risk estimates, the UNSCEARL and -LQ models, which have the most parameters in the parametric baseline, have the highest weights. For the models fitted to the PrestonL-baseline this trend cannot be observed. However, in this case, the number of parameters only differ minimally. Another property of the AIC is its dimensional inconsistency (Kashyap 1980), which means that the probability of AIC favouring an over-parametrised model does not tend to zero even as the data set size tends to infinity. In contrast to the AIC, the BIC is dimensionally consistent and excludes more complex models with higher number of parameters earlier if the dataset size is larger than 8 (ln(2) = 2.079 > 2). This behaviour can be observed in Table 5, where the highest weighted model in the model-averaged excess risk estimates is the one with the least number of parameters for every excess risk estimate using the BIC weights. The impact of these two information criteria is especially visible when the according model-averaged excess risk estimates based on the variable baselines are compared. Using these models, the number of fit parameters varies extremely, which results in very different weightings of the models due to the properties of the criteria described before. However, comparing the model-average excess risk estimates based on the PrestonL baseline, it can be observed that the BEIR model is the favoured model by both criteria. Of course, the weighting of models differs also in this case. Further, the BIC involves an asymptotic approximation and does not have an information-theoretic justification. However, from theoretical considerations of dimensional consistency the BIC appears to be the best method for model selection (Walsh 2007).

**Fig. 6:**
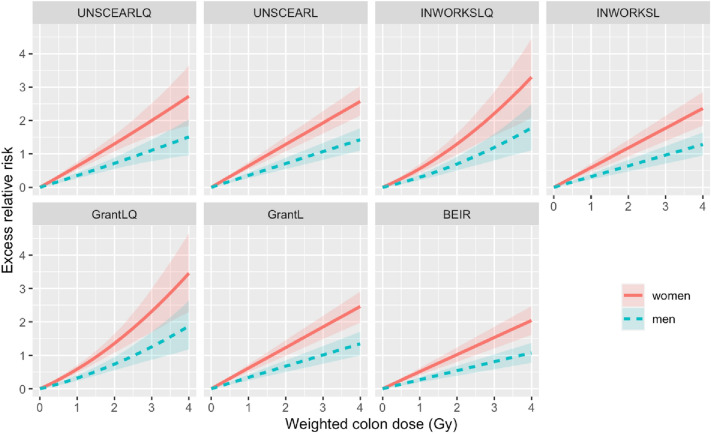
Sex-specific excess relative risks (ERRs) fitted with the PrestonL baseline as function of weighted colon dose for each model considered in this study, calculated at an attained age of 70 years and age at exposure of 30 years. Shaded areas represent 95% confidence intervals (CIs)

**Fig. 7:**
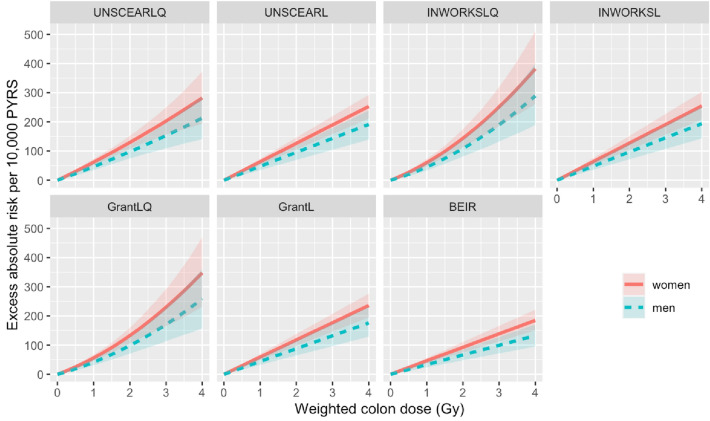
Sex-specific excess absolute risks (EARs) per 10^4^ PYRS fitted with the PrestonL baseline as function of weighted colon dose for each model considered in this study, calculated at an attained age of 70 years and age at exposure of 30 years. Shaded areas represent 95% confidence intervals **(**CIs)

**Fig. 8:**
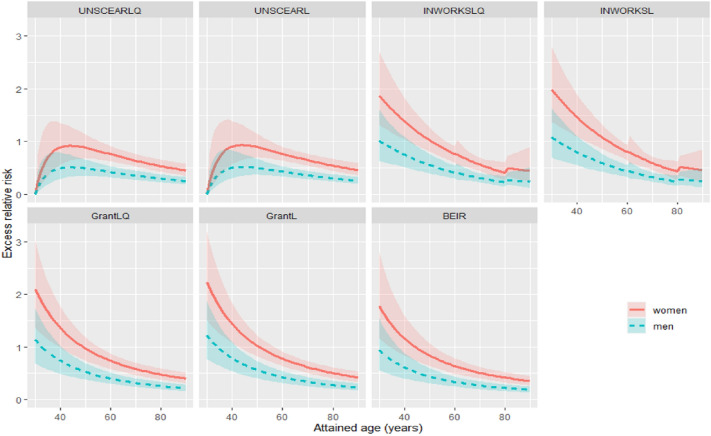
Sex-specific excess relative risks (ERRs) fitted with the PrestonL baseline as function of the attained age for each model considered in this study, assuming a weighted colon dose of 1 Gy. Shaded areas represent 95% confidence intervals (CIs)

**Fig. 9:**
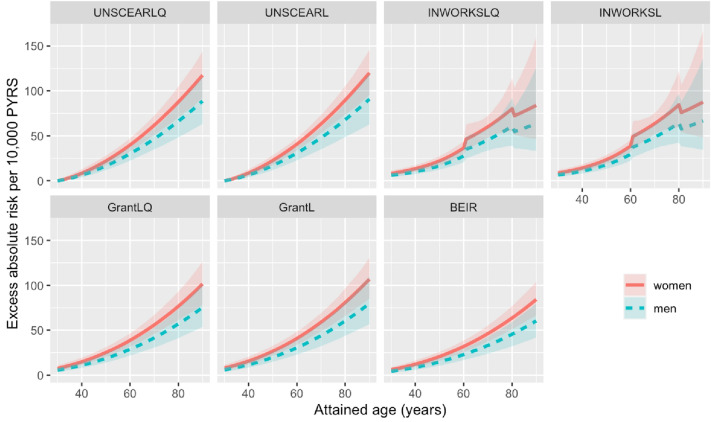
Sex-specific excess absolute risks (EARs) per 10^4^ PYRS fitted with the PrestonL baseline as function of the attained age for each radiation model considered in this study, assuming a weighted colon dose of 1 Gy. Shaded areas represent 95% confidence intervals (CIs)

Moreover, the ERR composite models fitted with the variable baseline hide an additional issue arising from the different baseline definition (parametric or by stratification) of the starting models considered. In particular, the extremely low weights assigned to BEIR VII Phase 2 (2006), Ozasa et al. (2012) and INWORKS-Leuraud et al. (2021) are a consequence of the modelling of the baseline rates, which are defined by stratification rather than being described by a parametric relation, common to the remaining models. These models can be clearly distinguished in the ERR panel of Fig. 6, clustered at higher AIC values, which consequently led to even smaller weights by several orders of magnitude (Table 4). In fact, the AIC and BIC methodologies consider the number of parameters present in each model, in addition to its deviance, penalising those models that are constituted by a larger number of parameters (Posada and Buckley 2004). However, when evaluating models whose baseline is defined by stratification, the AIC and BIC methods count each stratum as a parameter. While being of no concern when applying multi-model inference on models whose baselines are exclusively defended either by stratification or by parametrisation (such in the case of EARs), the AIC and BIC methodologies might suffer from limitations when MMI is applied to a set of models whose baselines have mixed parametric and stratified expressions. Referring to the cases reported in this study, for example, the number of parameters introduced by a parametrised baseline is between 12 and 23, far less that the 500–547 strata present in the stratified baselines. If one assumes similar model deviances, the relative risk models based on stratifications will then be greatly penalised in favour of the parametrised ones, merely because they are formulated in a different way.

As shown in the present study, this problem can be solved by setting a parametric definition of all the models’ baselines. With this method, the different radiation risk models can be compared. However, changing the baseline inevitably alters the initial models and makes them loose the level of detail typical of models with stratified baselines, which for example allows to better account for confounding factors. To circumvent such limitation of the weight assignment procedure based on AIC or BIC for total models with stratified baselines, other techniques can be explored. Future efforts could focus on devising techniques to adjust the obtained models’ weights and to normalise the number of parameters, so that the number of parameters in the baselines plays a minor role.

## Conclusions

In this work, 11 all-solid-cancer risk models have been refitted to the most recent LSS data (Grant et al. 2017), with the twofold objective of comparing the resulting ERRs and EARs and of applying model-averaging techniques to build a composite model to predict the risk of all solid cancer incidence related to ionising radiation exposures. The sex-specific estimates of the risk predicted by the models considering a variable baseline vary within 11% for ERR and within 16% for EAR, for a weighted colon dose of 1 Gy. Notwithstanding similar trends and similar order of magnitude of the risks predicted by the models considered, appreciable differences, induced by the different approach or by the dataset used for their development, can be noticed. Such differences should be recognised and taken into account whenever risk assessments on radiation-related incidence (or mortality) are performed. The multi-model inference techniques applied to the evaluated set of models yielded different composite models, depending on which information criteria and baseline parametrization was used. Considering BIC weights, the linear and linear-quadratic models from Grant et al. (2017) dominate when the models are fitted with the model-own baseline. The composite model for these models based on AIC weights is predominantly influenced by the linear and linear-quadratic models of UNSCEAR (2006). Fitting all excess risk models with the baseline from Preston et al. (2007), the BEIR model results to be the dominant model using AIC and BIC weighting. It is hence generally recommended to take model uncertainty into account in future risk analyses. As shown in other studies (Walsh 2007; Walsh and Kaiser 2011; Zhang and Townsend 2009), the AIC and BIC as a basis for model averaging constitute powerful tools that can be applied in the field of radiation epidemiology to enhance risk inference. However, in some instances, these information criteria show some limitations: for example, one disadvantage is the penalisation of models whose baseline is defined by stratification instead of parametrically. Whenever models with both parametric and stratified baselines are included in the same analysis, the assignment of weights is nevertheless problematic, even considering alternative approaches such as e.g. Pearson’s Chi squared, because they depend simultaneously on the goodness of fit and the number of parameters. Even though fitting the radiation risk models to the same parametric baseline provides a solution for comparison of the radiation risk models, a comparison of the total models is not possible without penalising the models with stratified baselines. Future efforts on this topic shall focus on investigating methodologies to address such problems, by, for example, adjust or re-normalise the number of parameters of the stratified models so that parametric ones are not favoured a priori, before applying the AIC or BIC.

### Electronic supplementary material

Below is the link to the electronic supplementary material.

Supplementary file1 (PDF 324 KB)

## Data Availability

The datafile used for this analysis is available on the RERF website: https://www.rerf.or.jp/en/library/dataen/lssinc07/

## Appendix 1

See Figs. 6, 7, 8, 9 and Tables

**Table 6:** List of the fitted risk coefficients for each model fitted with the variable baseline

Model	ERR parameters	EAR parameters
BEIR VII phase 2 (2006)	*α*_men_ = 0.29 ± 0.04; *α*_women_ = 0.55 ± 0.06 *ν* = − 1.43 ± 0.23 τ = − 0.35 ± 0.07	*α*_men_ = 35.6 ± 4.9; *α*_women_ = 48.6 ± 4.6 *ν* = 2.43 ± 0.21 *τ* = − 0.46 ± 0.06
Grant et al. (2017) Linear	*α* = 0.50 ± 0.04 *ν* = − 1.57 ± 0.24 *τ* = − 0.21 ± 0.05 *σ* = 0.29 ± 0.06	*α* = 53.3 ± 4.8 *ν* = 2.35 ± 0.21 *τ* = − 0.32 ± 0.05 *σ* = 0.14 ± 0.06
Grant et al. (2017) Linear-quadratic	*α* = 0.40 ± 0.06; *β* = 0.07 ± 0.04 *ν* = − 1.56 ± 0.24 *τ* = − 0.22 ± 0.05 *ν* = − 1.56 ± 0.24 *σ* = 0.29 ± 0.06	*α* = 41.3 ± 6.8; *β* = 9.2 ± 4.2 *ν* = 2.35 ± 0.21 *τ* = − 0.33 ± 0.05 *σ* = 0.14 ± 0.06
Ozasa et al. (2012) Linear	*α* = 0.52 ± 0.04 *ν* = − 1.47 ± 0.23 *τ* = − 0.25 ± 0.05 *σ* = 0.30 ± 0.06	*α* = 54.8 ± 4.8 *ν* = 2.41 ± 0.21 *τ* = − 0.33 ± 0.05 *σ* = 0.13 ± 0.06
Ozasa et al. (2012) Linear-quadratic	*α* = 0.45 ± 0.06; *β* = 0.05 ± 0.04 *ν* = − 1.46 ± 0.23 *τ* = − 0.25 ± 0.05 *σ* = 0.30 ± 0.06	*α* = 46.2 ± 6.8; *β* = 6.8 ± 4.2 *ν* = 2.41 ± 0.21 *τ* = − 0.34 ± 0.05 *σ* = 0.14 ± 0.06
Preston et al. (2007) Linear	*α* = 0.48 ± 0.04 *ν* = − 1.52 ± 0.24 *τ* = − 0.26 ± 0.05 *σ* = 0.29 ± 0.06	*α* = 51.5 ± 4.8 *ν* = 2.37 ± 0.21 *τ* = − 0.35 ± 0.05 *σ* = 0.14 ± 0.06
Preston et al. (2007) Linear-quadratic	*α* = 0.38 ± 0.06; *β* = 0.07 ± 0.04 *ν* = − 1.51 ± 0.24 *τ* = − 0.27 ± 0.05 *σ* = 0.30 ± 0.06	*α* = 39.7 ± 6.6; *β* = 9.0 ± 4.1 *ν* = 2.37 ± 0.21 *τ* = − 0.35 ± 0.05 *σ* = 0.15 ± 0.06
INWORKS-Leuraud (2021) Linear	*α* = 0.51 ± 0.05 *τ* = − 0.26 ± 0.05 *ν*_<60_ = − 0.28 ± 0.05; *ν*_>60,<80_ = − 0.17 ± 0.16; *ν*_>80_ = − 0.21 ± 0.18; *σ* = 0.30 ± 0.06	*α* = 59.6 ± 5.6 *τ* = − 0.32 ± 0.05 *ν*_<60_ = 0.51 ± 0.04; *ν*_>60,<80_ = 0.28 ± 0.14; *ν*_>80_ = 0.17 ± 0.16; *σ* = 0.12 ± 0.06
INWORKS-Leuraud (2021) Linear-quadratic	*α* = 0.44 ± 0.07; *β* = 0.05 ± 0.04 *τ* = − 0.26 ± 0.05 *ν*_<60_ = − 0.27 ± 0.04; *ν*_>60,<80_ = − 0.16 ± 0.16; *ν*_>80_ = − 0.20 ± 0.18; *σ* = 0.30 ± 0.06	*α* = 49.5 ± 7.6; *β* = 7.8 ± 4.6 *τ* = − 0.33 ± 0.05 *ν*_<60_ = 0.51 ± 0.04; *ν*_>60,<80_ = 0.28 ± 0.14; *ν*_>80_ = 0.18 ± 0.16; *σ* = 0.13 ± 0.06
UNSCEAR (2006) Linear	*α* = 0.38 ± 0.04 *σ* = 0.58 ± 0.12 *ε* = 0.86 ± 0.19 *ν* = − 2.76 ± 0.29	α = 51.1 ± 6.2 σ = 0.26 ± 0.12 ε = 1.13 ± 0.19 ν = 0.73 ± 0.24
UNSCEAR (2006) Linear-quadratic	*α* = 0.38 ± 0.06; *β* = − 0.004 ± 0.022 *σ* = 0.58 ± 0.12 *ε* = 0.86 ± 0.19 *ν* = − 2.76 ± 0.27	*α* = 51.1 ± 7.9; β = 0.03 ± 3.03 *σ* = 0.26 ± 0.12 *ε* = 1.12 ± 0.19 *ν* = 0.73 ± 0.24

The standard errors are also given

6,

**Table 7:** List of the fitted risk coefficients for each model fitted with the Preston et al. (2007) baseline

Model	ERR parameters	EAR parameters
BEIR VII phase 2 (2006)	*α*_men_ = 0.27 ± 0.04; *α*_women_ = 0.51 ± 0.06 *ν* = − 1.47 ± 0.24 *τ* = − 0.36 ± 0.07	*α*_men_ = 33.1 ± 4.8; *α*_women_ = 46.2 ± 4.6 *ν* = 2.39 ± 0.21 *τ* = − 0.47 ± 0.06
Grant et al. (2017) Linear	*α* = 0.48 ± 0.04 *ν* = − 1.52 ± 0.24 *τ* = − 0.26 ± 0.05 *σ* = 0.29 ± 0.06	*α* = 51.5 ± 4.8 *ν* = 2.37 ± 0.21 *τ* = − 0.35 ± 0.05 *σ* = 0.14 ± 0.06
Grant et al. (2017) Linear-quadratic	*α* = 0.38 ± 0.06; *β* = 0.07 ± 0.04 *ν* = − 1.51 ± 0.24 *τ* = − 0.27 ± 0.05 *σ* = 0.30 ± 0.06	*α* = 39.7 ± 6.6; β = 9.0 ± 4.1 *ν* = 2.37 ± 0.21 *τ* = − 0.35 ± 0.05 *σ* = 0.15 ± 0.06
INWORKS- Leuraud (2021) Linear	*α* = 0.46 ± 0.05 *τ* = − 0.26 ± 0.06 *ν*_<60_ = − 0.30 ± 0.05; *ν*_>60,<80_ = − 0.33 ± 0.16; *ν*_>80_ = − 0.13 ± 0.18; *σ* = 0.29 ± 0.06	*α* = 56.1 ± 5.6 *τ* = − 0.34 ± 0.05 *ν*_<60_ = 0.50 ± 0.04; *ν*_>60,<80_ = 0.28 ± 0.15; *ν*_>80_ = 0.16 ± 0.17; *σ* = 0.14 ± 0.06
INWORKS- Leuraud (2021) Linear-quadratic	*α* = 0.36 ± 0.06; *β* = 0.07 ± 0.04 *τ* = − 0.27 ± 0.06 *ν*_<60_ = − 0.30 ± 0.05; *ν*_>60,<80_ = − 0.32 ± 0.16; *ν*_>80_ = − 0.12 ± 0.18; *σ* = 0.30 ± 0.06	*α* = 42.4 ± 7.4; *β* = 10.4 ± 4.5 *τ* = − 0.34 ± 0.05 *ν*_<60_ = 0.50 ± 0.04; *ν*_>60,<80_ = 0.29 ± 0.15; *ν*_>80_ = 0.17 ± 0.17; *σ* = 0.14 ± 0.06
UNSCEAR (2006) Linear	*α* = 0.36 ± 0.04 *σ* = 0.60 ± 0.13 *ε* = 0.90 ± 0.20 *ν* = − 2.84 ± 0.27	*α* = 47.8 ± 6.2 *σ* = 0.28 ± 0.13 *ε* = 1.17 ± 0.20 *ν* = 0.66 ± 0.25
UNSCEAR (2006) Linear-quadratic	*α* = 0.34 ± 0.06; *β* = 0.009 ± 0.022 *σ* = 0.59 ± 0.13 *ε* = 0.91 ± 0.20 *ν* = − 2.84 ± 0.27	*α* = 44.5 ± 7.5; *β* = 2.19 ± 2.96 *σ* = 0.28 ± 0.13 *ε* = 1.18 ± 0.20 *ν* = 0.66 ± 0.25

The standard errors are also given

7.
