# Supplementary material for: Comparison and multi-model inference of excess risks models for radiation-related solid cancer
Source: Radiat Environ Biophys. 2023 Jan 21;62(1):17–34. doi: 10.1007/s00411-022-01013-0 (PMC9950237; doi:10.1007/s00411-022-01013-0)
Supplement: Supplementary file 1 — Supplementary file1 (PDF 324 KB) [file 411_2022_1013_MOESM1_ESM.pdf]

Supplementary information

Variable baseline

Model	ERR risk coefficients		EAR risk coefficients	
	males	females	males	females
**BEIR VII phase 2 (2006)**	-	-	${k_{1,s}}^{hiro} = 6.721$<br>${k_{1,s}}^{naga} = 3.577$<br>$k_{2,s} = -0.2284$<br>$k_{3,s} = -0.05104$<br>$k_{4,s} = 0.07936$<br>$k_{5,s} = -0.1515$<br>$k_{6,s} = 10.25$<br>$k_{7,s} = 3.364$<br>$k_{8,s} = -4.562$<br>$k_{9,s} = -8.884$<br>$k_{10,s} = 0$	${k_{1,s}}^{hiro} = 6.692$<br>${k_{1,s}}^{naga} = 3.563$<br>$k_{2,s} = -0.0573$<br>$k_{3,s} = 0.0002201$<br>$k_{4,s} = -0.004094$<br>$k_{5,s} = -0.1348$<br>$k_{6,s} = 0.4769$<br>$k_{7,s} = -2.041$<br>$k_{8,s} = 2.926$<br>$k_{9,s} = -5.308$<br>$k_{10,s} = 0$
**Grant et al. (2017) Linear**	$k_{1,s} = 5.255$<br>$k_{2,s} = 5.610$<br>$k_{3,s} = 0.1067$<br>$k_{4,s} = -12.44$<br>$k_{5,s} = 0.1509$<br>$c_{hiro} = -0.04212$<br>$c_{naga} = -0.1021$	$k_{1,s} = 4.499$<br>$k_{2,s} = 3.528$<br>$k_{3,s} = 0.1022$<br>$k_{4,s} = -3.611$<br>$k_{5,s} = 0.07248$<br>$c_{hiro} = -0.04212$<br>$c_{naga} = -0.1021$	$k_{1,s} = 5.265$<br>$k_{2,s} = 5.493$<br>$k_{3,s} = -0.3723$<br>$k_{4,s} = -11.49$<br>$k_{5,s} = 0.1522$<br>$c_{hiro} = -0.04453$<br>$c_{naga} = -0.1049$	$k_{1,s} = 4.496$<br>$k_{2,s} = 3.550$<br>$k_{3,s} = 0.2401$<br>$k_{4,s} = -3.916$<br>$k_{5,s} = 0.07065$<br>$c_{hiro} = -0.04453$<br>$c_{naga} = -0.1049$
**Grant et al. (2017) Linear-quadratic**	$k_{1,s} = 5.258$<br>$k_{2,s} = 5.607$<br>$k_{3,s} = 0.1124$<br>$k_{4,s} = -12.44$<br>$k_{5,s} = 0.1511$<br>$c_{hiro} = -0.04643$<br>$c_{naga} = -0.1065$	$k_{1,s} = 4.504$<br>$k_{2,s} = 3.524$<br>$k_{3,s} = 0.1093$<br>$k_{4,s} = -3.619$<br>$k_{5,s} = 0.07281$<br>$c_{hiro} = -0.04643$<br>$c_{naga} = -0.1065$	$k_{1,s} = 5.267$<br>$k_{2,s} = 5.490$<br>$k_{3,s} = -0.3570$<br>$k_{4,s} = -11.49$<br>$k_{5,s} = 0.1523$<br>$c_{hiro} = -0.04927$<br>$c_{naga} = -0.1097$	$k_{1,s} = 4.502$<br>$k_{2,s} = 3.545$<br>$k_{3,s} = 0.2438$<br>$k_{4,s} = -3.908$<br>$k_{5,s} = 0.07122$<br>$c_{hiro} = -0.04927$<br>$c_{naga} = -0.1097$
**Ozasa et al. (2012) Linear**	-	-	${k_{1,s}}^{hiro} = 6.831$<br>${k_{1,s}}^{naga} = 3.651$<br>$k_{2,s} = -0.2279$<br>$k_{3,s} = -0.05535$<br>$k_{4,s} = 0.08143$<br>$k_{5,s} = -0.1551$<br>$k_{6,s} = 10.65$<br>$k_{7,s} = 3.449$<br>$k_{8,s} = -4.889$<br>$k_{9,s} = -9.263$<br>$k_{10} = 0.09309$	${k_{1,s}}^{hiro} = 6.802$<br>${k_{1,s}}^{naga} = 3.637$<br>$k_{2,s} = -0.05618$<br>$k_{3,s} = -0.004399$<br>$k_{4,s} = -0.0006667$<br>$k_{5,s} = -0.1386$<br>$k_{6,s} = 0.7365$<br>$k_{7,s} = -2.040$<br>$k_{8,s} = 2.707$<br>$k_{9,s} = -5.506$<br>$k_{10} = 0.09309$
**Ozasa et al. (2012) Linear-quadratic**	-	-	${k_{1,s}}^{hiro} = 6.816$<br>${k_{1,s}}^{naga} = 3.663$<br>$k_{2,s} = -0.2276$<br>$k_{3,s} = -0.05509$<br>$k_{4,s} = 0.08106$<br>$k_{5,s} = -0.1549$<br>$k_{6,s} = 10.59$<br>$k_{7,s} = 3.415$	${k_{1,s}}^{hiro} = 6.787$<br>${k_{1,s}}^{naga} = 3.648$<br>$k_{2,s} = -0.05645$<br>$k_{3,s} = -0.004231$<br>$k_{4,s} = -0.0009220$<br>$k_{5,s} = -0.1379$<br>$k_{6,s} = 0.7617$<br>$k_{7,s} = -2.015$

			$k_{8,s} = -4.837$<br>$k_{9,s} = -9.285$<br>$k_{10} = 0.09314$	$k_{8,s} = 2.681$<br>$k_{9,s} = -5.500$<br>$k_{10} = 0.09314$
**Preston et al. (2007) Linear**	$k_{1,s} = 5.311$<br>$k_{2,s} = -0.01755$<br>$k_{3,s} = 5.549$<br>$k_{4,s} = 0.07918$<br>$k_{5,s} = -12.15$<br>$k_{6,s} = -0.1517$<br>$k_{7,s} = -0.01805$<br>$c_{hiro} = -0.05104$<br>$c_{naga} = -0.09698$	$k_{1,s} = 4.524$<br>$k_{2,s} = -0.01755$<br>$k_{3,s} = 3.513$<br>$k_{4,s} = 0.1135$<br>$k_{5,s} = -3.609$<br>$k_{6,s} = -0.07035$<br>$k_{7,s} = -0.008259$<br>$c_{hiro} = -0.05104$<br>$c_{naga} = -0.09698$	$k_{1,s} = 5.320$<br>$k_{2,s} = -0.01521$<br>$k_{3,s} = 5.428$<br>$k_{4,s} = -0.4096$<br>$k_{5,s} = -11.17$<br>$k_{6,s} = -0.1533$<br>$k_{7,s} = -0.01829$<br>$c_{hiro} = -0.05264$<br>$c_{naga} = -0.1015$	$k_{1,s} = 4.522$<br>$k_{2,s} = -0.01521$<br>$k_{3,s} = 3.536$<br>$k_{4,s} = 0.2595$<br>$k_{5,s} = -3.920$<br>$k_{6,s} = -0.06881$<br>$k_{7,s} = -0.009519$<br>$c_{hiro} = -0.05264$<br>$c_{naga} = -0.1015$
**Preston et al. (2007) Linear-quadratic**	$k_{1,s} = 5.315$<br>$k_{2,s} = -0.01961$<br>$k_{3,s} = 5.547$<br>$k_{4,s} = 0.08540$<br>$k_{5,s} = -12.16$<br>$k_{6,s} = -0.1521$<br>$k_{7,s} = -0.01801$<br>$c_{hiro} = -0.05597$<br>$c_{naga} = -0.09998$	$k_{1,s} = 4.524$<br>$k_{2,s} = -0.01961$<br>$k_{3,s} = 3.509$<br>$k_{4,s} = 0.1213$<br>$k_{5,s} = -3.617$<br>$k_{6,s} = -0.07093$<br>$k_{7,s} = -0.008213$<br>$c_{hiro} = -0.05597$<br>$c_{naga} = -0.09998$	$k_{1,s} = 5.324$<br>$k_{2,s} = -0.01739$<br>$k_{3,s} = 5.425$<br>$k_{4,s} = -0.3932$<br>$k_{5,s} = -11.18$<br>$k_{6,s} = -0.1535$<br>$k_{7,s} = -0.01822$<br>$c_{hiro} = -0.05798$<br>$c_{naga} = -0.1047$	$k_{1,s} = 4.528$<br>$k_{2,s} = -0.01739$<br>$k_{3,s} = 3.531$<br>$k_{4,s} = 0.2631$<br>$k_{5,s} = -3.913$<br>$k_{6,s} = -0.06963$<br>$k_{7,s} = -0.009379$<br>$c_{hiro} = -0.05798$<br>$c_{naga} = -0.1047$
**INWORKS-Leuraud (2021) Linear**	-	-	${k_{1,s}}^{hiro} = 6.762$<br>${k_{1,s}}^{naga} = 3.532$<br>$k_{2,s} = -0.2280$<br>$k_{3,s} = -0.05560$<br>$k_{4,s} = 0.08161$<br>$k_{5,s} = -0.1552$<br>$k_{6,s} = 10.40$<br>$k_{7,s} = 3.253$<br>$k_{8,s} = -4.673$<br>$k_{9,s} = -9.214$<br>$k_{10} = 0.09671$	${k_{1,s}}^{hiro} = 6.733$<br>${k_{1,s}}^{naga} = 3.519$<br>$k_{2,s} = -0.05653$<br>$k_{3,s} = -0.004850$<br>$k_{4,s} = -0.0003700$<br>$k_{5,s} = -0.138$<br>$k_{6,s} = 0.3086$<br>$k_{7,s} = -2.393$<br>$k_{8,s} = 3.080$<br>$k_{9,s} = -5.389$<br>$k_{10} = 0.09671$
**INWORKS-Leuraud (2021) Linear-quadratic**	-	-	${k_{1,s}}^{hiro} = 6.750$<br>${k_{1,s}}^{naga} = 3.550$<br>$k_{2,s} = -0.2277$<br>$k_{3,s} = -0.05529$<br>$k_{4,s} = 0.08119$<br>$k_{5,s} = -0.1550$<br>$k_{6,s} = 10.35$<br>$k_{7,s} = 3.226$<br>$k_{8,s} = -4.630$<br>$k_{9,s} = -9.239$<br>$k_{10} = 0.09652$	${k_{1,s}}^{hiro} = 6.720$<br>${k_{1,s}}^{naga} = 3.535$<br>$k_{2,s} = -0.05677$<br>$k_{3,s} = -0.004641$<br>$k_{4,s} = -0.0006456$<br>$k_{5,s} = -0.1376$<br>$k_{6,s} = 0.3541$<br>$k_{7,s} = -2.351$<br>$k_{8,s} = 3.036$<br>$k_{9,s} = -5.394$<br>$k_{10} = 0.09652$
**UNSCEAR (2006) Linear**	$k_0 = 4.879$<br>$k_1 = -0.3948$<br>$k_2 = 33.89$<br>$k_3 = 18.54$<br>$k_4 = -66.83$<br>$k_5 = -1.285$<br>$k_6 = -16.72$<br>$k_7 = -4.298$	$k_0 = 4.879$<br>$k_1 = -0.3948$<br>$k_2 = 33.89$<br>$k_3 = 18.54$<br>$k_4 = -66.83$<br>$k_5 = -1.285$<br>$k_6 = -16.72$<br>$k_7 = -4.298$	$k_0 = 4.879$<br>$k_1 = -0.4031$<br>$k_2 = 38.79$<br>$k_3 = 20.70$<br>$k_4 = -74.76$<br>$k_5 = -0.5757$<br>$k_6 = -19.47$<br>$k_7 - 5.047$	$k_0 = 4.879$<br>$k_1 = -0.4031$<br>$k_2 = 38.79$<br>$k_3 = 20.70$<br>$k_4 = -74.76$<br>$k_5 = -0.5757$<br>$k_6 = -19.47$<br>$k_7 - 5.047$

	$k_8 = -4.346$<br>$k_9 = -0.2640$<br>$k_{10} = -0.3490$<br>$k_{11} = 2.260$<br>$k_{12} = 1.907$<br>$k_{13} = -0.1516$<br>$k_{14} = -0.007650$<br>$k_{15} = -11.39$<br>$k_{16} = 34.93$<br>$k_{17} = -9.948$<br>$k_{18} = -5.418$<br>$k_{19} = 7.929$<br>$k_{20} = 1.013$<br>$k_{21} = 10.02$<br>$k_{22} = -7.531$	$k_8 = -4.346$<br>$k_9 = -0.2640$<br>$k_{10} = -0.3490$<br>$k_{11} = 2.260$<br>$k_{12} = 1.907$<br>$k_{13} = -0.1516$<br>$k_{14} = -0.007650$<br>$k_{15} = -11.39$<br>$k_{16} = 34.93$<br>$k_{17} = -9.948$<br>$k_{18} = -5.418$<br>$k_{19} = 7.929$<br>$k_{20} = 1.013$<br>$k_{21} = 10.02$<br>$k_{22} = -7.531$	$k_8 = -5.043$<br>$k_9 = -0.3018$<br>$k_{10} = -0.3475$<br>$k_{11} = 2.391$<br>$k_{12} = 1.913$<br>$k_{13} = -0.1590$<br>$k_{14} = -0.009421$<br>$k_{15} = -12.27$<br>$k_{16} = 38.77$<br>$k_{17} = -12.57$<br>$k_{18} = -5.819$<br>$k_{19} = 9.168$<br>$k_{20} = 1.329$<br>$k_{21} = 11.32$<br>$k_{22} = -8.458$	$k_8 = -5.043$<br>$k_9 = -0.3018$<br>$k_{10} = -0.3475$<br>$k_{11} = 2.391$<br>$k_{12} = 1.913$<br>$k_{13} = -0.1590$<br>$k_{14} = -0.009421$<br>$k_{15} = -12.27$<br>$k_{16} = 38.77$<br>$k_{17} = -12.57$<br>$k_{18} = -5.819$<br>$k_{19} = 9.168$<br>$k_{20} = 1.329$<br>$k_{21} = 11.32$<br>$k_{22} = -8.458$
**UNSCEAR (2006) Linear-quadratic**	$k_0 = 4.879$<br>$k_1 = -0.3948$<br>$k_2 = 33.9$<br>$k_3 = 18.56$<br>$k_4 = -66.83$<br>$k_5 = -1.285$<br>$k_6 = -16.72$<br>$k_7 = -4.298$<br>$k_8 = -4.347$<br>$k_9 = -0.2640$<br>$k_{10} = -0.3489$<br>$k_{11} = 2.260$<br>$k_{12} = 1.907$<br>$k_{13} = -0.1516$<br>$k_{14} = -0.007632$<br>$k_{15} = -11.39$<br>$k_{16} = 34.93$<br>$k_{17} = -9.947$<br>$k_{18} = -5.421$<br>$k_{19} = 7.928$<br>$k_{20} = 1.012$<br>$k_{21} = 10.03$<br>$k_{22} = -7.531$	$k_0 = 4.879$<br>$k_1 = -0.3948$<br>$k_2 = 33.9$<br>$k_3 = 18.56$<br>$k_4 = -66.83$<br>$k_5 = -1.285$<br>$k_6 = -16.72$<br>$k_7 = -4.298$<br>$k_8 = -4.347$<br>$k_9 = -0.2640$<br>$k_{10} = -0.3489$<br>$k_{11} = 2.260$<br>$k_{12} = 1.907$<br>$k_{13} = -0.1516$<br>$k_{14} = -0.007632$<br>$k_{15} = -11.39$<br>$k_{16} = 34.93$<br>$k_{17} = -9.947$<br>$k_{18} = -5.421$<br>$k_{19} = 7.928$<br>$k_{20} = 1.012$<br>$k_{21} = 10.03$<br>$k_{22} = -7.531$	$k_0 = 4.879$<br>$k_1 = -0.4030$<br>$k_2 = 38.79$<br>$k_3 = 20.70$<br>$k_4 = -74.76$<br>$k_5 = -0.5759$<br>$k_6 = -19.47$<br>$k_7 = -5.047$<br>$k_8 = -5.043$<br>$k_9 = -0.3018$<br>$k_{10} = -0.3475$<br>$k_{11} = 2.391$<br>$k_{12} = 1.913$<br>$k_{13} = -0.1590$<br>$k_{14} = -0.009420$<br>$k_{15} = -12.27$<br>$k_{16} = 38.77$<br>$k_{17} = -12.57$<br>$k_{18} = -5.819$<br>$k_{19} = 9.168$<br>$k_{20} = 1.329$<br>$k_{21} = 11.32$<br>$k_{22} = -8.457$	$k_0 = 4.879$<br>$k_1 = -0.4030$<br>$k_2 = 38.79$<br>$k_3 = 20.70$<br>$k_4 = -74.76$<br>$k_5 = -0.5759$<br>$k_6 = -19.47$<br>$k_7 = -5.047$<br>$k_8 = -5.043$<br>$k_9 = -0.3018$<br>$k_{10} = -0.3475$<br>$k_{11} = 2.391$<br>$k_{12} = 1.913$<br>$k_{13} = -0.1590$<br>$k_{14} = -0.009420$<br>$k_{15} = -12.27$<br>$k_{16} = 38.77$<br>$k_{17} = -12.57$<br>$k_{18} = -5.819$<br>$k_{19} = 9.168$<br>$k_{20} = 1.329$<br>$k_{21} = 11.32$<br>$k_{22} = -8.457$

Table S1 Baseline risk coefficients for the risk models with variable baselines shown in Table 1.

GrantL_ERR	OzasaL_ERR	UNSCEARL_ERR	BEIR_ERR	PrestonL_ERR
0.0019121	0.0019591	0.0019142	0.0015425	0.0019192
0.0028172	0.0025118	-0.0040985	0.0011067	0.0025041
0.0010535	0.0012425	-0.0025694	-1.23E-04	0.0012728
-0.00027541	-0.00027309	0.0051413	0.0015545	-0.00028855
0.0028172	0.0025118	-0.0040985	0.0011067	0.0025041
0.055733	0.054973	0.01542	0.0033078	0.056079
-0.0050049	-0.0049726	0.0022646	0.0017045	-0.0052276
0.001626	0.0014949	-0.00021747	0.0024171	0.0016861
0.0010535	0.0012425	-0.0025694	-1.23E-04	0.0012728
-0.0050049	-0.0049726	0.0022646	0.0017045	-0.0052276
0.0026142	0.0027922	0.037532	0.054892	0.00297
-0.0003091	-0.0003296	-0.033901	-0.0065866	-0.00036101
-0.00027541	-0.00027309	0.0051413	0.0015545	-0.00028855
0.001626	0.0014949	-0.00021747	0.0024171	0.0016861
-0.0003091	-0.0003296	-0.033901	-0.0065866	-0.00036101
0.0034451	0.0032586	0.073778	0.0044252	0.0034819

Table S2 Covariance matrices in vector form for the linear Grant, Ozasa UNSCEAR, BEIR and Preston ERR models. The sequence of the covariance parameters corresponds to the sequence of the risk coefficients listed in Table 6.

GrantL_EAR	OzasaL_EAR	UNSCEARL_EAR	BEIR_EAR	PrestonL_EAR
22.774	22.608	38.514	24.395	22.893
0.28962	0.26416	-0.62423	11.374	0.27048
0.11565	0.11837	-0.25086	1.36E-01	0.12955
-0.11256	0.10847	0.74396	0.15813	-0.11248
0.28962	0.26416	-0.62423	11.374	0.27048
0.043991	0.04237	0.015102	21.344	0.044088
-0.0046461	-0.0044328	0.00056722	0.0017545	-0.0046801
-0.0023572	-0.0021469	-0.0051991	0.19311	-0.0023384
0.11565	0.11837	-0.25086	1.36E-01	0.12955
-0.0046461	-0.0044328	0.00056722	0.0017545	-0.0046801
0.0025869	0.0024931	0.034646	0.042913	0.0027303
-0.00013213	-0.00014914	-0.029708	-0.0060679	-0.00017246
-0.11256	0.10847	0.74396	0.15813	-0.11248
-0.0023572	-0.0021469	-0.0051991	0.19311	-0.0023384
-0.00013213	-0.00014914	-0.029708	-0.0060679	-0.00017246
0.0038723	0.003672	0.055909	0.0038737	0.0039216

Table S3 Covariance matrices in vector form for the linear Grant, Ozasa UNSCEAR, BEIR and Preston EAR models. The sequence of the covariance parameters corresponds to the sequence of the risk coefficients listed in Table 6.

GRANTLQ_ ERR	OzasaLQ_ ERR	UNSCEARLQ _ERR	PrestonLQ_ ERR	INWORKSLQ _ERR	INWORKSL _ERR
0.0040742	0.004059	0.0033207	0.0038235	0.0043322	0.0023802
-0.0018214	-0.0017793	-0.00084585	-0.0016365	-0.0016934	0.0013492
0.0020964	0.0019559	-0.0045372	0.0018211	0.0012094	0.00090365
0.00089787	0.0010693	-0.0026697	0.0010796	0.00074438	0.00065502
-0.00043997	-0.00043184	0.0051721	-0.00043798	0.00040787	-0.0031547
-0.0018214	-0.0017793	-0.00084585	-0.0016365	-0.0029469	-0.0001826
0.0015246	0.0014986	0.00050769	0.0013902	-0.0003544	0.0013492
0.00052964	0.00044036	0.0002702	0.00050671	-0.0016934	0.0029212
0.00014288	0.00012429	6.55E-05	0.00016628	0.0014536	-0.00087369
0.00013806	0.0001352	-2.49E-05	0.00012695	0.00011972	-0.0017489
0.0020964	0.0019559	-0.0045372	0.0018211	0.00012108	-0.0020698
0.00052964	0.00044036	0.0002702	0.00050671	0.00018193	-0.00029277
0.055934	0.054188	0.015516	0.056274	-0.00018093	0.00090365
-0.0050595	-0.0049478	0.0022828	-0.005276	0.00014186	-0.00087369
0.0016833	0.0015234	-0.00021328	0.0017417	0.0012094	0.0024209
0.00089787	0.0010693	-0.0026697	0.0010796	0.00011972	0.0016205
0.00014288	0.00012429	6.55E-05	0.00016628	0.0029522	-0.0011459
-0.0050595	-0.0049478	0.0022828	-0.005276	-0.00088068	0.00036841
0.0026728	0.0027786	0.037474	0.0030225	-0.0017699	0.00065502
-0.00031119	-0.00032205	-0.033838	-0.0003614	-0.0020997	-0.0017489
-0.00043997	-0.00043184	0.0051721	-0.00043798	-0.0002914	0.0016205
0.00013806	0.0001352	-2.49E-05	0.00012695	0.00074438	0.025835
0.0016833	0.0015234	-0.00021328	0.0017417	0.00012108	-0.00059224
-0.00031119	-0.00032205	-0.033838	-0.0003614	-0.00088068	-0.0001569
0.0034493	0.0032499	0.073698	0.003483	0.0024262	-0.0031547
				0.0016344	-0.0020698
				-0.0011468	-0.0011459
				0.00037452	-0.00059224
				0.00040787	0.033905
				0.00018193	-0.00044954
				-0.0017699	-0.0001826
				0.0016344	-0.00029277
				0.025994	0.00036841
				-0.00057915	-0.0001569
				-0.00013641	-0.00044954
				-0.0029469	0.0032825
				-0.00018093	
				-0.0020997	
				-0.0011468	
				-0.00057915	
				0.034096	
				-0.00043741	
				-0.0003544	
				0.00014186	
				-0.0002914	
				0.00037452	
				-0.00013641	
				-0.00043741	
				0.0032852	

Table S4 Covariance matrices in vector form for the linear quadratic Grant, Ozasa UNSCEAR, INWORKS, Preston and linear INWORKS ERR models. The sequence of the covariance parameters corresponds to the sequence of the risk coefficients listed in Table 6.

GRANTLQ_ EAR	OzasaLQ_ EAR	UNSCEARLQ _EAR	PrestonLQ_ EAR	INWORKSLQ _EAR	INWORKSL _EAR
45.906	45.829	61.635	44.193	57.216	30.814
-20.474	-20.255	-14.554	-19.104	-23.526	0.13463
0.25094	0.23191	-0.66889	0.2332	0.1202	0.081006
0.096574	0.10397	-0.26629	0.10901	0.071754	0.10709
-0.10978	-0.11101	0.77605	-0.10858	0.085261	-0.28051
-20.474	-20.255	-14.554	-19.104	-0.26257	-0.11782
17.998	17.608	9.1629	16.958	-0.11916	0.13463
0.030261	0.026269	0.028069	0.029943	-23.526	0.0025466
0.018487	0.01341	9.67E-03	0.019164	20.78	-0.00082212
0.00034634	0.0036596	-2.01E-02	-0.00062666	0.013949	-0.00134
0.25094	0.23191	-0.66889	0.2332	0.0074518	-0.0017238
0.030261	0.026269	0.028069	0.029943	0.017365	-0.00011058
0.044988	0.043035	0.015191	0.045102	-0.019686	0.081006
-0.0047374	-0.0044879	0.0005976	-0.0047683	0.0031077	-0.00082212
-0.0022791	-0.0020975	-0.0052622	-0.0022585	0.1202	0.0017477
0.096574	0.10397	-0.26629	0.10901	0.013949	0.0014461
0.018487	0.01341	9.67E-03	0.019164	0.0025999	-0.00052842
-0.0047374	-0.0044879	0.0005976	-0.0047683	-0.00083348	-0.00043496
0.0026648	0.0025373	0.03466	0.0028047	-0.0013672	0.10709
-0.00013993	-0.00015254	-0.029734	-0.00018103	-0.0017676	-0.00134
-0.10978	-0.11101	0.77605	-0.10858	-0.00011535	0.0014461
0.00034634	0.0036596	-2.01E-02	-0.00062666	0.071754	0.019686
-0.0022791	-0.0020975	-0.0052622	-0.0022585	0.0074518	-0.00046651
-0.00013993	-0.00015254	-0.029734	-0.00018103	-0.00083348	-0.00050285
0.003882	0.0036817	0.055962	0.0039303	0.0017721	-0.28051
				0.0014687	-0.0017238
				-0.0005519	-0.00052842
				-0.00042435	-0.00046651
				0.085261	0.025555
				0.017365	-0.00033612
				-0.0013672	-0.11782
				0.0014687	-0.00011058
				0.020153	-0.00043496
				-0.00050949	-0.00050285
				-0.00050099	-0.00033612
				-0.26257	0.0036769
				-0.019686	
				-0.0017676	
				-0.0005519	
				-0.00050949	
				0.026216	
				-0.00033291	
				-0.11916	
				0.0031077	
				-0.00011535	
				-0.00042435	
				-0.00050099	
				-0.00033291	
				0.003688	

Table S5 Covariance matrices in vector form for the linear quadratic Grant, Ozasa UNSCEAR, INWORKS, Preston and linear INWORKS EAR models. The sequence of the covariance parameters corresponds to the sequence of the risk coefficients listed in Table 6.

Constant baseline

Model	ERR risk coefficients		EAR risk coefficients	
	males	females	males	females
**BEIR VII phase 2 (2006)**	$k_{1,s} = 5.314$<br>$k_{2,s} = -0.01649$<br>$k_{3,s} = 5.546$<br>$k_{4,s} = 0.08275$<br>$k_{5,s} = -12.13$<br>$k_{6,s} = -0.1529$<br>$k_{7,s} = -0.01929$<br>$c_{hiro} = -0.05104$<br>$c_{naga} = -0.09861$	$k_{1,s} = 4.527$<br>$k_{2,s} = -0.01649$<br>$k_{3,s} = 3.503$<br>$k_{4,s} = 0.1078$<br>$k_{5,s} = -3.556$<br>$k_{6,s} = -0.07154$<br>$k_{7,s} = -0.01031$<br>$c_{hiro} = -0.05104$<br>$c_{naga} = -0.09861$	$k_{1,s} = 5.323$<br>$k_{2,s} = -0.01422$<br>$k_{3,s} = 5.424$<br>$k_{4,s} = -0.4085$<br>$k_{5,s} = -11.14$<br>$k_{6,s} = -0.1549$<br>$k_{7,s} = -0.01952$<br>$c_{hiro} = -0.05246$<br>$c_{naga} = -0.1031$	$k_{1,s} = 4.526$<br>$k_{2,s} = -0.01422$<br>$k_{3,s} = 3.528$<br>$k_{4,s} = 0.2543$<br>$k_{5,s} = -3.865$<br>$k_{6,s} = -0.07160$<br>$k_{7,s} = -0.01184$<br>$c_{hiro} = -0.05246$<br>$c_{naga} = -0.1031$
**Grant et al. (2017) Linear**	$k_{1,s} = 5.311$<br>$k_{2,s} = -0.01755$<br>$k_{3,s} = 5.549$<br>$k_{4,s} = 0.07918$<br>$k_{5,s} = -12.15$<br>$k_{6,s} = -0.1517$<br>$k_{7,s} = -0.01805$<br>$c_{hiro} = -0.05104$<br>$c_{naga} = -0.09698$	$k_{1,s} = 4.524$<br>$k_{2,s} = -0.01755$<br>$k_{3,s} = 3.513$<br>$k_{4,s} = 0.1135$<br>$k_{5,s} = -3.609$<br>$k_{6,s} = -0.07035$<br>$k_{7,s} = -0.008259$<br>$c_{hiro} = -0.05104$<br>$c_{naga} = -0.09698$	$k_{1,s} = 5.320$<br>$k_{2,s} = -0.01521$<br>$k_{3,s} = 5.428$<br>$k_{4,s} = -0.4096$<br>$k_{5,s} = -11.17$<br>$k_{6,s} = -0.1533$<br>$k_{7,s} = -0.01829$<br>$c_{hiro} = -0.05264$<br>$c_{naga} = -0.1015$	$k_{1,s} = 4.522$<br>$k_{2,s} = -0.01521$<br>$k_{3,s} = 3.536$<br>$k_{4,s} = 0.2595$<br>$k_{5,s} = -3.920$<br>$k_{6,s} = -0.06881$<br>$k_{7,s} = -0.009519$<br>$c_{hiro} = -0.05264$<br>$c_{naga} = -0.1015$
**Grant et al. (2017) Linear-quadratic**	$k_{1,s} = 5.315$<br>$k_{2,s} = -0.01961$<br>$k_{3,s} = 5.547$<br>$k_{4,s} = 0.08540$<br>$k_{5,s} = -12.16$<br>$k_{6,s} = -0.1521$<br>$k_{7,s} = -0.01801$<br>$c_{hiro} = -0.05597$<br>$c_{naga} = -0.09998$	$k_{1,s} = 4.524$<br>$k_{2,s} = -0.01961$<br>$k_{3,s} = 3.509$<br>$k_{4,s} = 0.1213$<br>$k_{5,s} = -3.617$<br>$k_{6,s} = -0.07093$<br>$k_{7,s} = -0.008213$<br>$c_{hiro} = -0.05597$<br>$c_{naga} = -0.09998$	$k_{1,s} = 5.324$<br>$k_{2,s} = -0.01739$<br>$k_{3,s} = 5.425$<br>$k_{4,s} = -0.3932$<br>$k_{5,s} = -11.18$<br>$k_{6,s} = -0.1535$<br>$k_{7,s} = -0.01822$<br>$c_{hiro} = -0.05798$<br>$c_{naga} = -0.1047$	$k_{1,s} = 4.528$<br>$k_{2,s} = -0.01739$<br>$k_{3,s} = 3.531$<br>$k_{4,s} = 0.2631$<br>$k_{5,s} = -3.913$<br>$k_{6,s} = -0.06963$<br>$k_{7,s} = -0.009379$<br>$c_{hiro} = -0.05798$<br>$c_{naga} = -0.1047$
**INWORKS-Leuraud (2021) Linear**	$k_{1,s} = 5.313$<br>$k_{2,s} = -0.01750$<br>$k_{3,s} = 5.580$<br>$k_{4,s} = 0.1342$<br>$k_{5,s} = -12.38$<br>$k_{6,s} = -0.1518$<br>$k_{7,s} = -0.01813$<br>$c_{hiro} = -0.05127$<br>$c_{naga} = -0.09737$	$k_{1,s} = 4.525$<br>$k_{2,s} = -0.01750$<br>$k_{3,s} = 3.571$<br>$k_{4,s} = 0.2236$<br>$k_{5,s} = -4.013$<br>$k_{6,s} = -0.07039$<br>$k_{7,s} = -0.008365$<br>$c_{hiro} = -0.05127$<br>$c_{naga} = -0.09737$	$k_{1,s} = 5.318$<br>$k_{2,s} = -0.01502$<br>$k_{3,s} = 5.414$<br>$k_{4,s} = -0.4168$<br>$k_{5,s} = -11.03$<br>$k_{6,s} = -0.1533$<br>$k_{7,s} = -0.01827$<br>$c_{hiro} = -0.05257$<br>$c_{naga} = -0.1015$	$k_{1,s} = 4.520$<br>$k_{2,s} = -0.01502$<br>$k_{3,s} = 3.504$<br>$k_{4,s} = 0.2236$<br>$k_{5,s} = -3.642$<br>$k_{6,s} = -0.06894$<br>$k_{7,s} = -0.009560$<br>$c_{hiro} = -0.05257$<br>$c_{naga} = -0.1015$
**INWORKS-Leuraud (2021) Linear-quadratic**	$k_{1,s} = 5.316$<br>$k_{2,s} = -0.01952$<br>$k_{3,s} = 5.575$<br>$k_{4,s} = 0.1357$<br>$k_{5,s} = -12.37$<br>$k_{6,s} = -0.1522$<br>$k_{7,s} = -0.01808$<br>$c_{hiro} = -0.05608$<br>$c_{naga} = -0.1003$	$k_{1,s} = 4.530$<br>$k_{2,s} = -0.01952$<br>$k_{3,s} = 3.563$<br>$k_{4,s} = 0.2241$<br>$k_{5,s} = -3.997$<br>$k_{6,s} = -0.07094$<br>$k_{7,s} = -0.008308$<br>$c_{hiro} = -0.05608$<br>$c_{naga} = -0.1003$	$k_{1,s} = 5.322$<br>$k_{2,s} = -0.01736$<br>$k_{3,s} = 5.411$<br>$k_{4,s} = -0.4016$<br>$k_{5,s} = -11.04$<br>$k_{6,s} = -0.1535$<br>$k_{7,s} = -0.01820$<br>$c_{hiro} = -0.05824$<br>$c_{naga} = -0.1049$	$k_{1,s} = 4.527$<br>$k_{2,s} = -0.01736$<br>$k_{3,s} = 3.502$<br>$k_{4,s} = 0.2292$<br>$k_{5,s} = -3.664$<br>$k_{6,s} = -0.06977$<br>$k_{7,s} = -0.009402$<br>$c_{hiro} = -0.05824$<br>$c_{naga} = -0.1049$
**UNSCEAR (2006)**	$k_{1,s} = 5.311$<br>$k_{2,s} = -0.01780$	$k_{1,s} = 4.524$<br>$k_{2,s} = -0.01780$	$k_{1,s} = 5.320$<br>$k_{2,s} = -0.01555$	$k_{1,s} = 4.522$<br>$k_{2,s} = -0.01555$

**Linear**	$k_{3,s} = 5.535$<br>$k_{4,s} = 0.04477$<br>$k_{5,s} = -12.11$<br>$k_{6,s} = -0.1521$<br>$k_{7,s} = -0.01759$<br>$c_{hiro} = -0.05165$<br>$c_{naga} = -0.09748$	$k_{3,s} = 3.476$<br>$k_{4,s} = 0.04714$<br>$k_{5,s} = -3.443$<br>$k_{6,s} = -0.07087$<br>$k_{7,s} = -0.007893$<br>$c_{hiro} = -0.05165$<br>$c_{naga} = -0.09748$	$k_{3,s} = 5.417$<br>$k_{4,s} = -0.4326$<br>$k_{5,s} = -11.15$<br>$k_{6,s} = -0.1537$<br>$k_{7,s} = -0.01794$<br>$c_{hiro} = -0.05317$<br>$c_{naga} = -0.1020$	$k_{3,s} = 3.511$<br>$k_{4,s} = 0.2233$<br>$k_{5,s} = -3.854$<br>$k_{6,s} = -0.06996$<br>$k_{7,s} = -0.008887$<br>$c_{hiro} = -0.05317$<br>$c_{naga} = -0.1020$
**UNSCEAR (2006) Linear-quadratic**	$k_{1,s} = 5.311$<br>$k_{2,s} = -0.01818$<br>$k_{3,s} = 5.535$<br>$k_{4,s} = 0.04621$<br>$k_{5,s} = -12.11$<br>$k_{6,s} = -0.1522$<br>$k_{7,s} = -0.01758$<br>$c_{hiro} = -0.05262$<br>$c_{naga} = -0.09810$	$k_{1,s} = 4.525$<br>$k_{2,s} = -0.01818$<br>$k_{3,s} = 3.476$<br>$k_{4,s} = 0.04877$<br>$k_{5,s} = -3.445$<br>$k_{6,s} = -0.07098$<br>$k_{7,s} = -0.007890$<br>$c_{hiro} = -0.05262$<br>$c_{naga} = -0.09810$	$k_{1,s} = 5.321$<br>$k_{2,s} = -0.01621$<br>$k_{3,s} = 5.416$<br>$k_{4,s} = -0.4277$<br>$k_{5,s} = -11.15$<br>$k_{6,s} = -0.1538$<br>$k_{7,s} = -0.01793$<br>$c_{hiro} = -0.05487$<br>$c_{naga} = -0.1031$	$k_{1,s} = 4.524$<br>$k_{2,s} = -0.01621$<br>$k_{3,s} = 3.510$<br>$k_{4,s} = 0.2250$<br>$k_{5,s} = -3.854$<br>$k_{6,s} = -0.07020$<br>$k_{7,s} = -0.008859$<br>$c_{hiro} = -0.05487$<br>$c_{naga} = -0.1031$

Table S6: Baseline risk coefficients for the risk models fitted to the PrestonL baseline shown in Table 1.

GrantL_ERR	UNSCEARL_ERR	BEIR_ERR	GrantL_EAR	UNSCEARL_EAR	BEIR_EAR
0.0019192	0.0018314	0.0014774	22.893	38.14	23.385
0.0025041	-0.004106	0.0010721	0.27048	-0.64872	11.502
0.0012728	-0.0025565	1.38E-04	0.12955	-0.28466	1.50E-01
-0.00028855	0.0050803	0.0015352	-0.11248	0.79878	0.1592
0.0025041	-0.004106	0.0010721	0.27048	-0.64872	11.502
0.056079	0.016496	0.0031054	0.044088	0.016465	21.357
-0.0052276	0.0021747	0.0017779	-0.0046801	0.00084092	0.012827
0.0016861	0.00017851	0.0023407	-0.0023384	-0.0060674	0.20019
0.0012728	-0.0025565	-1.38E-04	0.12955	-0.28466	1.50E-01
-0.0052276	0.0021747	0.0017779	-0.0046801	0.00084092	0.012827
0.00297	0.03903	0.056854	0.0027303	0.038064	0.044578
-0.00036101	-0.034656	-0.0069126	-0.00017246	-0.032853	-0.006284
-0.00028855	0.0050803	0.0015352	-0.11248	0.79878	0.1592
0.0016861	0.00017851	0.0023407	-0.0023384	-0.0060674	0.20019
-0.00036101	-0.034656	-0.0069126	-0.00017246	-0.032853	-0.006284
0.0034819	0.075291	0.0045441	0.0039216	0.0607	0.0041002

Table S7 Covariance matrices in vector form for the linear Grant, UNSCEAR and BEIR ERR and EAR models fitted to the PrestonL baseline. The sequence of the covariance parameters corresponds to the sequence of the risk coefficients listed in Table 7.

GRANTLQ_ERR	UNSCEARLQ_ERR	INWORKSLQ_ERR	INWORKSL_ERR
0.0038235	0.0030549	0.0038236	0.0022295
-0.0016365	-0.00075968	-0.0014466	0.0013105
0.0018211	-0.0043502	0.0011233	0.00092884
0.0010796	-0.0026214	0.00071268	0.0013364
-0.00043798	0.0050103	0.0010138	-0.002932
-0.0016365	-0.00075968	-0.0025732	-0.00020503
0.0013902	0.00047276	-0.00036071	0.0013105
0.00050671	0.0001413	-0.0014466	0.0030756
0.00016628	2.86E-05	0.0012785	-0.00094646
0.00012695	5.54E-05	0.00016221	-0.0015662
0.0018211	-0.0043502	0.00015958	-0.0020831
0.00050671	1.41E-04	0.00023292	-0.00032787
0.056274	0.016626	-0.00032299	0.00092884
-0.005276	0.0022148	0.00013021	-0.00094646
0.0017417	0.00017282	0.0011233	0.0025476
0.0010796	-0.0026214	0.00016221	0.0023145
0.00016628	2.86E-05	0.0031338	-0.0011629
-0.005276	0.0022148	-0.00095797	0.00037587
0.0030225	0.039216	-0.0016029	0.0013364
-0.0003614	-0.034837	-0.0021392	-0.0015662
-0.00043798	0.0050103	-0.00032826	0.0023145
0.00012695	5.54E-05	0.00071268	0.025274
0.0017417	0.00017282	0.00015958	-0.0011254
-0.0003614	-0.034837	-0.00095797	-3.58E-06
0.003483	0.075514	0.0025566	-0.002932
		0.0023207	-0.0020831
		-0.0011861	-0.0011629
		0.00038553	-0.0011254
		0.0010138	0.031283
		0.00023292	-0.00047586
		-0.0016029	-0.00020503
		0.0023207	-0.00032787
		0.025914	0.00037587
		-0.0011872	-3.58E-06
		3.76E-06	-0.00047586
		-0.0025732	0.0034884
		-0.00032299	
		-0.0021392	
		-0.0011861	
		-0.0011872	
		0.032119	
		-0.00046724	
		-0.00036071	
		0.00013021	
		-0.00032826	
		0.00038553	
		3.76E-06	
		-0.00046724	
		0.0034895	

Table S8 Covariance matrices in vector form for the linear quadratic Grant, UNSCEAR, INWORKS and linear INWORKS ERR models fitted to the PrestonL baseline. The sequence of the covariance parameters corresponds to the sequence of the risk coefficients listed in Table 7.

GRANTLQ_EAR	UNSCEARLQ_EAR	INWORKSLQ_EAR	INWORKSL_EAR
44.193	56.88	54.528	30.908
-19.104	-12.82	-22.081	0.14635
0.2332	-0.65192	0.12418	0.081713
0.10901	-0.29474	0.069122	0.10552
-0.10858	0.80493	0.079601	-0.28817
-19.104	-12.82	-0.24845	-0.12304
16.958	8.7753	-0.11551	0.14635
0.029943	0.0010587	-22.081	0.0027986
0.019164	3.45E-03	20.186	-0.00088303
-0.00062666	1.01E-03	0.021206	-0.0014856
0.2332	-0.65192	0.010048	-0.0019071
0.029943	0.0010587	0.021128	-0.00013432
0.045102	0.016575	-0.040002	0.081713
-0.0047683	0.00088669	-0.0027167	-0.00088303
-0.0022585	-0.0061237	0.12418	0.0018275
0.10901	-0.29474	0.021206	0.0015619
0.019164	0.0034548	0.0028871	-0.00054882
-0.0047683	8.87E-04	-0.00090138	-0.00047938
0.0028047	0.038448	-0.0015313	0.10552
-0.00018103	-0.033264	-0.0019807	-0.0014856
-0.10858	0.80493	-0.00014304	0.0015619
-0.00062666	0.0010103	0.069122	0.021187
-0.0022585	-6.12E-03	0.010048	-0.00044429
-0.00018103	-0.033264	-0.00090138	-0.00055555
0.0039303	0.061454	0.0018669	-0.28817
		0.0016055	-0.0019071
		-0.00058882	-0.00054882
		-0.00046134	-0.00044429
		0.079601	0.029281
		0.021128	-0.0003565
		-0.0015313	-0.12304
		0.0016055	-0.00013432
		0.021984	-0.00047938
		-0.00053052	-0.00055555
		-0.00055567	-0.0003565
		-0.24845	0.0039323
		-0.040002	
		-0.0019807	
		-0.00058882	
		-0.00053052	
		0.030452	
		-0.00036033	
		-0.11551	
		-0.0027167	
		-0.00014304	
		-0.00046134	
		-0.00055567	
		-0.00036033	
		0.0039427	

Table S9 Covariance matrices in vector form for the linear quadratic Grant, UNSCEAR, INWORKS and linear INWORKS EAR models fitted to the PrestonL baseline. The sequence of the covariance parameters corresponds to the sequence of the risk coefficients listed in Table 7.
